# A metabolic shift to the serine pathway induced by lipids fosters epigenetic reprogramming in nontransformed breast cells

**DOI:** 10.1126/sciadv.ads9182

**Published:** 2025-03-21

**Authors:** Mariana Bustamante Eduardo, Gannon Cottone, Curtis W. McCloskey, Shiyu Liu, Flavio R. Palma, Maria Paula Zappia, Abul B.M.M.K. Islam, Peng Gao, Joel Setya, Saya Dennis, Hongyu Gao, Qian Zhang, Xiaoling Xuei, Yuan Luo, Jason Locasale, Marcelo G. Bonini, Rama Khokha, Maxim V. Frolov, Elizaveta V. Benevolenskaya, Navdeep S. Chandel, Seema A. Khan, Susan E. Clare

**Affiliations:** ^1^ Department of Surgery, Feinberg School of Medicine, Northwestern University, Chicago, IL, USA.; ^2^ Princess Margaret Cancer Centre, University Health Network, Toronto, Ontario, Canada.; ^3^ Department of Pharmacology and Cancer Biology, Duke University, Durham, NC, USA.; ^4^ Department of Medicine/Division of Hematology Oncology, Feinberg School of Medicine, Northwestern University, Chicago, IL, USA.; ^5^ Department of Biochemistry and Molecular Genetics, University of Illinois at Chicago, Chicago, IL, USA.; ^6^ Robert H. Lurie Cancer Center Metabolomics Core, Feinberg School of Medicine, Northwestern University, Chicago, IL, USA.; ^7^ Department of Preventive Medicine, Northwestern University, Chicago, IL, USA.; ^8^ Center for Medical Genomics, Indiana University School of Medicine, Indianapolis, IN, USA.; ^9^ Robert H. Lurie Cancer Center of Northwestern University, Chicago, IL, USA.; ^10^ Division of Pulmonary and Critical Care Medicine, Department of Medicine, Northwestern University, Chicago, IL, USA.; ^11^ Department of Biochemistry and Molecular Genetics, Northwestern University, Chicago, IL, USA.

## Abstract

Lipid metabolism and the serine, one-carbon, glycine (SOG) and methionine pathways are independently and significantly correlated with estrogen receptor–negative breast cancer (ERneg BC). Here, we propose a link between lipid metabolism and ERneg BC through phosphoglycerate dehydrogenase (PHGDH), the rate-limiting enzyme in the de novo serine pathway. We demonstrate that the metabolism of the paradigmatic medium-chain fatty acid octanoic acid leads to a metabolic shift toward the SOG and methionine pathways. PHGDH plays a role in both the forward direction, contributing to the production of S-adenosylmethionine, and the reverse direction, generating the oncometabolite 2-hydroxyglutarate, leading to epigenomic reprogramming and phenotypic plasticity. The methionine cycle is closely linked to the transsulfuration pathway. Consequently, we observe that the shift increases the antioxidant glutathione, which mitigates reactive oxygen species (ROS), enabling survival of a subset of cells that have undergone DNA damage. These metabolic changes contribute to several hallmarks of cancer.

## Teaser

ERneg BCs originates from metabolic mechanisms involving the SOG and methionine pathways.

## INTRODUCTION

Breast cancer (BC)–related mortality is decreasing due to more effective therapies and early detection. However, BC incidence continues to increase globally (*1*). This underscores the deficiency of current preventive strategies, particularly those that protect against estrogen receptor–negative BC (ERneg BC) (*2*). To achieve subtype-specific cancer prevention, using agents that intercept or prevent carcinogenesis (*3*), an understanding of the etiology of the different subtypes of BC is needed. For example, premenopausal obesity, African ancestry, *BRCA1* carrier status, and certain germline nucleotide variants are associated with a higher frequency of ERneg BC (*4*–*7*). Nevertheless, the strongest risk factors for BC, other than germline mutations in tumor suppressor genes, are in the breast and include breast epithelial atypia and increased mammographic density (*8*, *9*).

We have focused on examining the in-breast microenvironment to identify factors that promote ERneg BC and that may be disrupted for prevention. Since studies of metachronous contralateral BC (CBC) show a similarity in the ER status of the CBC to the index primary (*10*, *11*), we used the contralateral, unaffected breast (CUB) of patients with unilateral BC as a model to discover potential markers of subtype-specific risk. We analyzed gene expression profiles of epithelial cells from CUBs and identified a lipid metabolism gene signature enriched in the CUBs of women with ERneg BC (*12*, *13*). To better understand lipid metabolism in the breast, we studied the effect of fatty acids on nontransformed breast epithelial cells: Metabolic flux analysis based on RNA sequencing predicted an increased flux through several metabolic reactions including those involved in serine, one carbon (1C), and glycine (SOG); methionine; and in reactions producing endogenous antioxidants. Moreover, fatty acid exposure resulted in profound changes in chromatin packing density, chromatin accessibility, histone posttranslational modifications, and gene expression (*14*).

Consulting metabolic reaction network reconstructions, the most likely hypothesis to account for the increase in SOG and methionine cycle metabolic flux is the shunting of metabolic activity from glycolysis to the de novo serine pathway. To determine how this occurs, its consequences, and to further study the effects of fatty acids on the normal breast, we have performed metabolomics, epigenomic profiling, and single-cell RNA sequencing (scRNA-seq) on human breast epithelial cell lines and primary human tissue-derived breast microstructures. We show that the metabolism of medium-chain fatty acids in preference to glucose and glutamine rewires cellular metabolism away from glycolysis and toward the SOG and methionine pathways, increasing S-adenosylmethionine (SAM), glutathione (GSH), and 2-hydroxyglutarate (2-HG) levels, leading to DNA damage, survival of specific cell types, and epigenetic fostered phenotypic plasticity.

## RESULTS

### Octanoic acid shifts metabolism toward the de novo serine pathway

**Fig. 1.**
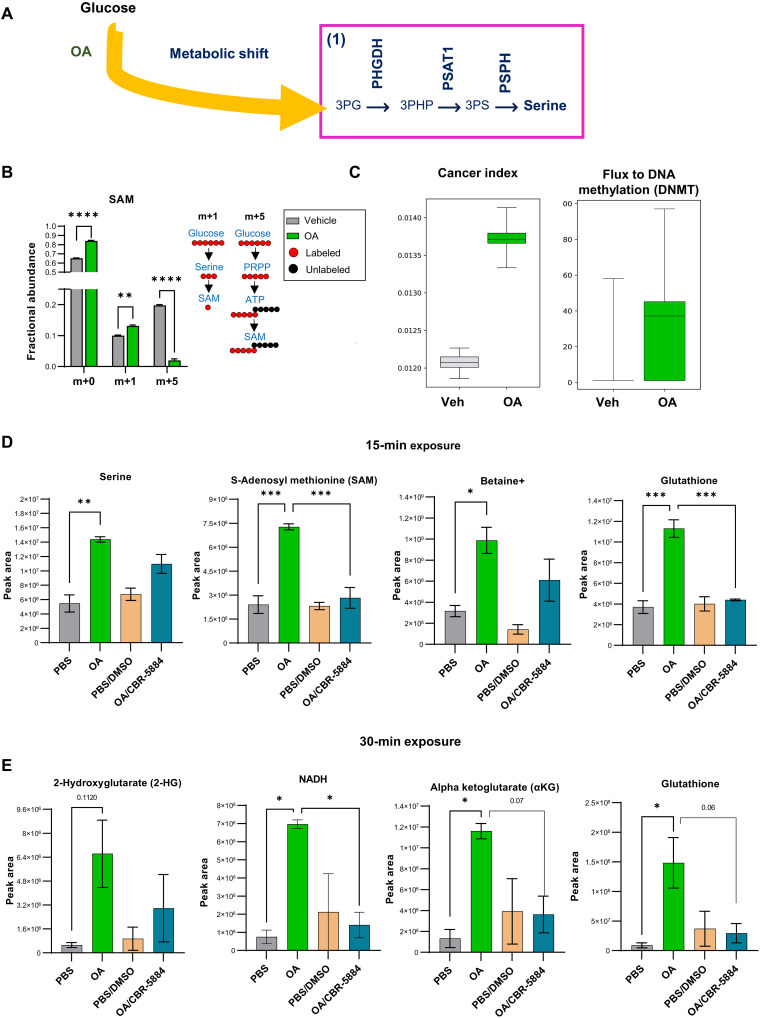
Metabolic shift. (**A**) Metabolism of OA results in a metabolic shift toward the de novo serine pathway. (**B**) Fractional abundance of SAM isotopologues. **P < 0.01, ****P < 0.0001 [two-way analysis of variance (ANOVA) with Tukey test, *n* = 3, mean with SD]. Four hours of labeling. Right: Schematic of derivation and contribution of carbon atoms in SAM synthesis. (**C**) ^13^C metabolic flux calculations. Cancer index was calculated as the lactate ratio. (**D**) Measurement of serine, SAM, betaine+, GSH, 2-HG, NADH, and αKG after 15- and 30-min (**E**) exposure to PBS (vehicle), OA, PBS plus DMSO (vehicle), and OA plus CBR-5884. *P < 0.05, **P < 0.01, ***P < 0.001 (two-way ANOVA with Tukey test, *n* = 3, mean with SEM).

We previously showed that the medium-chain fatty acid octanoic acid (OA) drives flux through many metabolic reactions including those involved in SOG and methione pathways. This was based on RNA sequencing–inferred metabolic flux (*14*). To test whether, in the presence of OA, carbons from glucose are being diverted into the SOG and methionine pathways, thereby increasing the production of the main methyl donor SAM, we performed U^13^C-glucose tracing in MCF-10A cells exposed to OA. Twenty-four-hour exposure to OA increased fractional abundance of SAM M+1 through the serine pathway (Fig. 1B); in addition, it decreased phosphoribosyl pyrophosphate (PRPP) M+5 and the fractional abundance of adenosine triphosphate (ATP) M+5-7 (fig. S1A). Metabolic flux analysis of the U^13^C tracing revealed that upon OA exposure, glucose uptake, glycolysis, and pentose phosphate pathway flux decreased (fig. S1B), and 1C-THF was redirected to the methionine cycle increasing flux to DNA methylation (Fig. 1C). OA-treated cells had an increased cancer index (defined as the lactate ratio) (Fig. 1C), noncanonical Tricarboxylic Acid Cycle (TCA) index, and TCA index (fig. S1C).

We hypothesized that the first and rate-limiting step in the de novo serine pathway, which is catalyzed by phosphoglycerate dehydrogenase (PHGDH), is key to these observations. In the forward direction, PHGDH participates in SOG and methionine pathways that produce SAM, while in the reverse direction PHGDH produces the oncometabolite 2-HG (*15*). We next performed targeted metabolomics in MCF-10A cells exposed to OA in the presence or absence of the PHGDH inhibitor CBR-5884. OA exposure increased production of serine, SAM, GSH, and betaine at 15 min, while 2-HG, GSH, NADH [reduced form of nicotinamide adenine dinucleotide (oxidized form) (NAD^+^)], and α ketoglutarate (αKG) production increased after 30 min of OA exposure (Fig. 1, D and E). Blocking PHGDH with CBR-5884 prevented these increases (Fig. 1, D and E).

These results show rewiring cellular metabolism away from glycolysis and toward the SOG and methionine pathways through fatty acid exposure increases the production of endogenous antioxidants such as GSH, the main methyl donor SAM, and the oncometabolite 2-HG.

### OA-induced SAM increases global DNA methylation

To assess the effect of the increased concentration of SAM (Fig. 1D) on DNA methylation, genome-wide DNA methylation profiling was performed using the Illumina Infinium Methylation EPIC Beadchip array in MCF-10A cells exposed to vehicle, OA, or OA plus CBR-5884. While the median CpG methylation level was higher upon OA treatment, the median CpG methylation level in OA-treated cells and cells exposed to OA plus CBR-5884 was similar (fig. S2). However, differential methylation analysis comparing vehicle- and OA-treated cells revealed no statistically significant differences after correction for multiple testing, suggesting that PHGDH regulates gene expression programs through a DNA methylation–independent mechanism.

### OA-induced SAM and 2-HG production drives a neural-like transcriptional program through histone methylation

Given the robust induction of the main methyl donor SAM (Fig. 1D) with OA treatment, we postulated that OA exposure would consequently alter the histone methylation landscape. Similarly, the increase of 2-HG (Fig. 1E), acting as an inhibitor of αKG-dependent dioxygenases such as histone demethylases (*16*), would also contribute to the increased methylation by blocking demethylation. To assess the methylation landscape upon OA exposure (*14*), we performed CUT&RUN for H3K27me3 and H3K4me3. These are histone marks associated with repression of gene expression and with transcriptionally active chromatin, respectively.

CUT&RUN for H3K27me3 revealed 12 peaks differentially enriched in control [false discovery rate (FDR) < 0.05] and associated with increased gene expression in OA (fig. S3, A and B, and table S1A). Among them were stem cell markers LGR6 (up-regulated by OA, log_2_FC = 1.9) (*14*), a stem cell marker also associated with ERneg BC, and PLAG1 (up-regulated by OA, log_2_FC = 2.8) (*14*). With regard to the increase in LGR6 expression, the percentage of ALDH^+^ cells (a marker of stem cells) increased from 15% to 30% (*P* < 0.01) after OA exposure (fig. S3C).

**Fig. 2.**
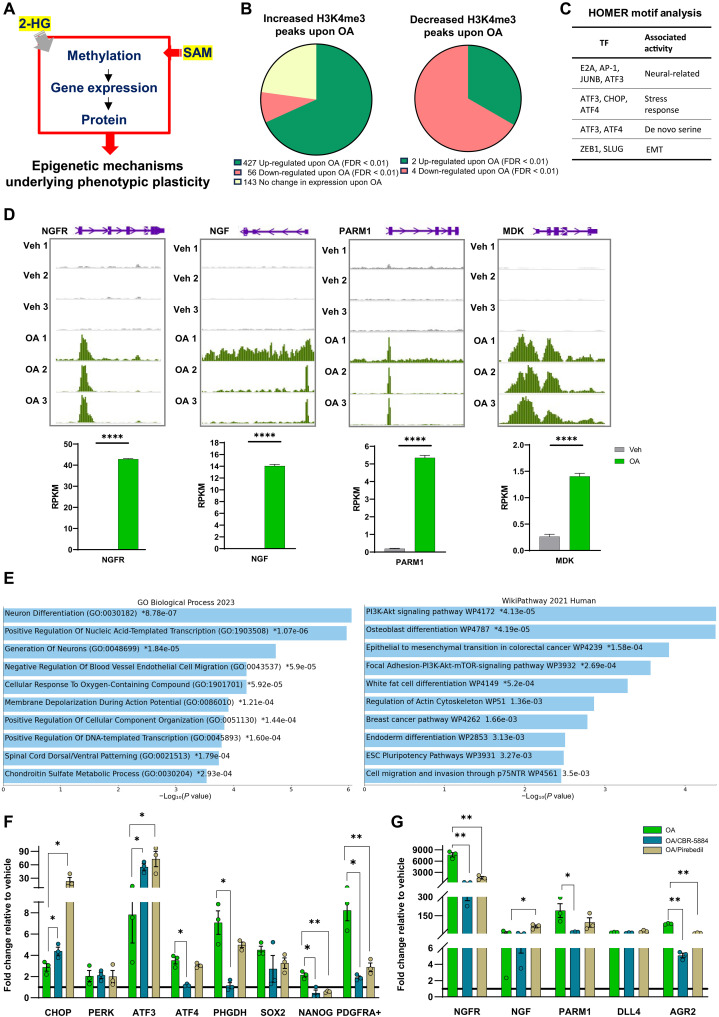
Epigenetic reprogramming. (**A**) Increases in 2-HG and SAM lead to epigenomic reprogramming. (**B**) Pie chart depicting the distribution of OA-associated H3K4me3 peaks (FDR < 0.01) at OA-modulated genes (FDR < 0.01). (**C**) Table depicting relevant transcription factor (TF) motifs identified with HOMER in increased H3K4me3 peaks and their associated activity. (**D**) Histograms showing H3K4me3 occupancy at NGFR, NGF, PARM1, and MDK in vehicle (gray tracks) and OA (green tracks). The peaks are visualized with the WashU Epigenome Browser. The bottom panel shows boxplots of RNA expression reads per kilobase of transcript per million mapped reads (RPKM) values. ****P < 0.0001. (**E**) Gene Ontology (GO) Biological Process 2021 and WikiPathway 2021 classifications for genes overexpressed upon OA treatment and associated with increased H3K4me3 peaks. (**F**) Boxplots showing the expression of OA-induced genes upon OA, OA plus CBR-5884, and OA plus Piribedil measured by qPCR. +Gene with enriched H3K4me3 peak upon OA. (**G**) Boxplots showing the expression of OA-induced neural-related genes with enriched H3K4me3 peaks. *P < 0.05, **P < 0.01 (multiple unpaired *t* test, mean with SEM).

H3K4me3 CUT&RUN identified 661 differential peaks significantly enriched in OA-treated cells (table S1B). Seventy-three percent of H3K4me3 OA-associated peaks were in regulatory regions of OA-induced genes (FDR < 0.01) (*14*) (Fig. 2B). Homer motif analysis revealed an overrepresentation of binding sites for transcription factors (*P* < 0.05) associated with Epithelial-Mesenchymal Transition (EMT) (ZEB1, SLUG), neural functions (E2A, AP1, JUNB), neuronal injury (ATF3), serine pathway (ATF3, ATF4), and stress response (CHOP, ATF3, ATF4) (Fig. 2C). Examples of significantly up-regulated genes concurrent with H3K4me3 enrichment upon OA exposure are shown in Fig. 2D. We used Enrichr (https://maayanlab.cloud/Enrichr/) (*17*) to perform pathway analysis of OA-induced genes with increased H3K4me3 peaks. Among the overrepresented pathways were those involved in EMT, neural-related pathways, embryonic stem cell pluripotency, cell migration and invasion, and BC (Fig. 2E). To determine whether inhibition of enzymatic reactions in the de novo serine pathway and/or H3K4 trimethylation block the OA-induced changes in gene expression, MCF-10A cells were exposed to OA in the presence or absence of the PHGDH inhibitor CBR-5884 or the histone methyltransferase inhibitor Piribedil, and gene expression was quantified by real-time quantitative PCR (RT-qPCR). Blocking PHGDH significantly prevented OA-induced expression of *ATF4*, while increasing *ATF3* and *CHOP* and having no effect on *PERK* (Fig. 2F). Piribedil significantly induced the expression of *ATF3*, *ATF4*, and *CHOP* similarly without affecting *PERK* (Fig. 2F). OA induced the expression of *PHGDH*, which was prevented by CBR-5884 (Fig. 2F). *NANOG* and *PDGFRA* induction was prevented by both CBR-5884 and Piribedil (Fig. 2F). The expression of H3K4me3-controlled genes *NGFR*, *PARM1*, and *AGR2* was prevented by CBR-5884 and Piribedil (Fig. 2G). The increase of *NGF* was prevented by CBR-5884, while Piribedil induced its expression further (Fig. 2G). To investigate the metabolic flip in the SOG and methionine pathways in tissue-derived breast microstructures, we inhibited the de novo serine pathway using the U.S. Food and Drug Administration–approved clofazimine, which has been shown to inhibit PSPH, which catalyzes the third step of the de novo serine pathway (*18*). RT-qPCR analysis (fig. S4A) showed that in microstructures from BRCA1 wild-type individuals, clofazimine significantly blocked OA-induced expression of H3K4me3-controlled genes *AGR2* and *PARM1*, with a trend toward reduced *PHGDH* and *NTRK2* expression. In BRCA1 mutation carriers, clofazimine significantly prevented OA-induced expression of H3K4me3 *NTRK2*, with a trend toward reduced *PHGDH* and *PARM1* expression (fig. S4B). Overall, these results confirm that many of the changes in gene expression are driven by the metabolic flip toward the SOG and the methionine cycle and occur as a result of epigenetic reprogramming secondary to the increase of H3K4me3.

### OA-exposed MCF-10A cells adopt a neural-like phenotype when grown on poly-d-lysine/laminin–coated plates

Given the strong signal of neural-like differentiation noted above (Fig. 2E), which we had also observed in the bulk RNA sequencing data (*14*), we sought to determine whether OA-exposed MCF-10A cells adopt a neural phenotype in cell culture. Poly-d-lysine and laminin are used in neural cell culture to promote cell attachment, growth, and differentiation. Therefore, OA-exposed MCF-10A cells were cultured on poly-d-lysine/laminin–coated plates (fig. S5A). Culture of MCF-10A in the presence of OA clearly results in the switch to a neural phenotype complete with the outgrowth of neurites and a cell body that is polygonal. Neurite outgrowth was also observed when cells grew out from breast microstructures (the first step in establishing primary epithelial cultures) into poly-d-lysine and laminin plates in the presence of OA (fig. S5, B and C). Additionally, OA exposure of the ERneg basal-like cell line MCF10ADCIS.com led to the adoption of a neural phenotype (fig. S5D) and to the increased expression of OA-responsive genes (fig. S6).

### Cells deploy antioxidant defenses to control the concentration of reactive oxygen species

**Fig. 3.**
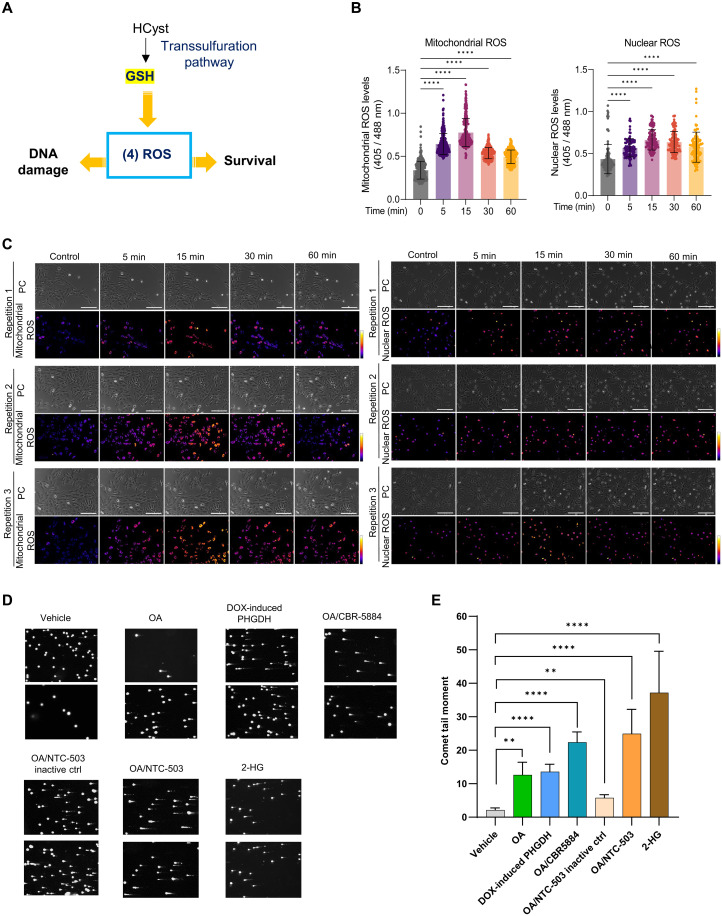
Antioxidant defenses. (**A**) Cells deploy antioxidant defenses to control the concentration of ROS. (**B**) Quantification of mito-roGFP2-Orp1 and nls-roGFP2-Orp1 monitoring mitochondrial and nuclear redox state in MCF-10A cells, respectively. Ratio of emission at 405 and 488 nm obtained as a response of OA exposure. ****P < 0.0001 (ordinary one-way ANOVA with Tukey test, mean with SD). (**C**) Representative pictures of mito-roGFP2-Orp1 and nls-roGFP2-Orp1 monitoring mitochondrial and nuclear redox state in MCF-10A cells. Scale bar, 200 μm. (**D**) Comet tail moment for control MCF-10A (vehicle), doxycycline (DOX)-induced PHGDH, MCF-10A in the presence of OA ± PHGDH inhibitors (CBR-5884 or NTC-503), or NTC-503 inactive control and 2-HG. (**E**) Quantification of (D). **P < 0.01, ****P < 0.0001 (two-way ANOVA with Tukey test, mean with SEM).

Cells deploy antioxidant defenses such as GSH, NADH, and αKG upon OA exposure (Fig. 1, D and E). In addition, we have previously shown that medium- and long-chain polyunsaturated fatty acid exposure increases mitochondrial catalase and ALDH1L1 (*14*), which may also have antioxidant roles. We postulated that OA exposure increases reactive oxygen species (ROS) due to enhanced fatty acid oxidation (*19*) while simultaneously driving compensatory GSH production through increased methionine cycle flux/transsulfuration pathway activity. Therefore, we monitored ROS-induced redox changes live for 60 min in MCF-10A cells transduced with the ORP1-roGFP2 redox reporter vectors that localize to either the mitochondria or nucleus. This revealed a significant increase of mitochondrial and nuclear ROS after 5 min of OA exposure (Fig. 3, B and C). Mitochondrial and nuclear ROS levels remained significantly higher after 60 min of exposure. Mitochondrial and nuclear ROS peaked (*P* < 0.0001) after 15 min with OA and then slowly decreased, consistent with the timing of GSH production (Fig. 3, B and C). Nuclear ROS decreased much more slowly than mitochondrial ROS (Fig. 3, B and C). Thus, OA exposure results in the deployment of antioxidant defenses to mitigate increased concentrations of ROS.

### OA exposure leads to DNA breaks

OA exposure leads to a significant increase of ROS (Fig. 3, B and C) and d-2-HG (fig. S7, A and B), an enantiomer produced by PHGDH. DNA damage is one of the harmful effects of ROS (*20*). Among the αKG-dependent dioxygenases inhibited by 2-HG are KDM 4A/B, which are required for homologous recombination (HR) repair (*21*). These two facts led us to hypothesize that OA-exposed cells evidence unrepaired DNA damage. We performed an alkaline comet assay in MCF-10A cells exposed to OA, OA plus PHGDH inhibitors, and 2-HG, and in MCF-10A cells overexpressing *PHGDH* (Fig. 3, D and E). Alkaline comet assay showed that OA and exogenous 2-HG significantly increased comet tails. However, adding PHGDH inhibitor CBR-5884 or NTC-503 did not decrease comet tails. This is likely a consequence of increased ROS due to the reduction of GSH upon PHGDH inhibition. To test this, we performed a GSH rescue experiment. OA exposure led to a decrease in cell viability, with a more pronounced reduction observed when the de novo serine pathway was inhibited, likely due to lowered GSH levels. Cell viability was rescued by the addition of N-acetylcysteine (fig. S7C). Significantly increased comet tails were also observed in MCF-10A cells expressing doxycycline-induced *PHGDH* likely due to the overexpression of this gene, which leads to the increase of d-2-HG (fig. S7, D and E). To determine whether OA-induced d-2-HG increase leads to HR deficiency, we used a luciferase-based reporter assay that measures HR; the construct enables the restoration of luciferase expression by HR following a cut within a luciferase gene. Since 900 μM 2-HG has been shown to impair HR (*21*), we treated the cells with exogenous 2-HG at this concentration as a control (fig. S7F). While exogenous 2-HG impaired HR, there was no significant difference between vehicle and OA. It is likely that ROS is mostly responsible for the DNA damage and that a higher 2-HG concentration is required to impair HR. It is also clear that mitigation of ROS is insufficient to prevent DNA damage as indicated by the comet tails.

### OA affects the cellular composition of breast microstructures

**Fig. 4.**
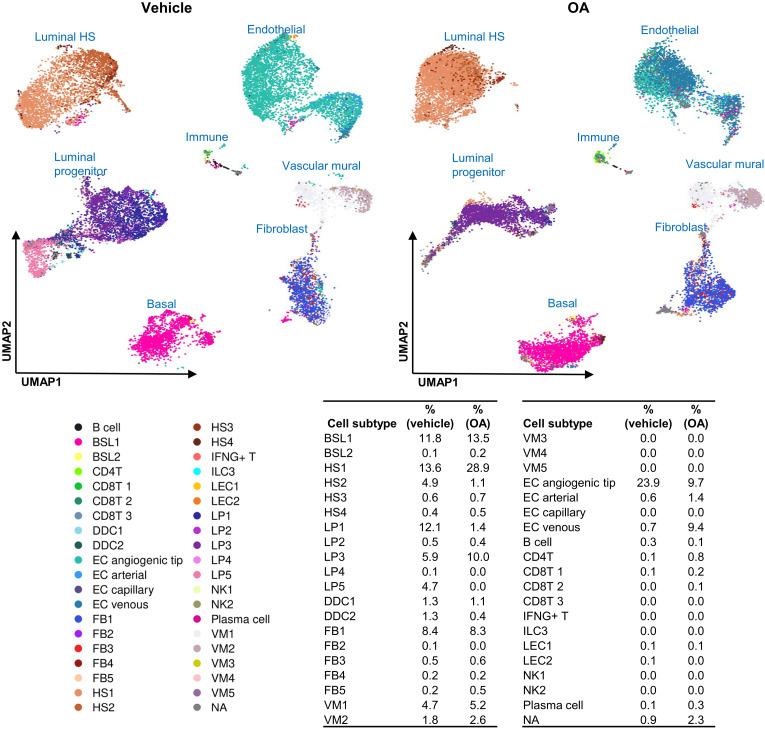
Single-cell landscape of vehicle- and OA-treated tissue-derived breast microstructures. Uniform Manifold Approximation and Projection (UMAP) plot of 36,904 cells identified a total of 25 different cell clusters. Cell subtypes defined by Reed *et al.* (*65*). Table shows cell subtype proportions.

Given the robust effects of OA on transcriptional programs via histone methylation in MCF-10A cells (Fig. 2), we next sought to profile the effects of OA at a more granular level. To explore the effects of fatty acids in human breast tissue ex vivo, we exposed human breast microstructures derived from reduction mammoplasty tissue from 12 donors to vehicle or OA and measured OA-induced gene expression (*14*) by RT-qPCR. There were six donors from wild-type individuals and six from BRCA1 mutation carriers. Most of the selected genes, including neural-related genes, were induced by OA in tissue-derived microstructures from both wild-type donors and BRCA1 mutation carriers (fig. S8, A and B). Because breast microstructures retain the original architectural integrity as well as the different cell subtypes of the breast environment (*22*), we further explored the effects of OA by scRNA-seq. For this, breast microstructures from two wild-type donors were exposed to vehicle and OA for 24 hours. Single-cell transcriptomic profiles for 36,904 cells and 41,445 genes were selected for the analysis. A total of 25 clusters were identified including epithelial, fibroblasts, endothelial cells, and immune cells (Fig. 4). Within the epithelial compartment, we have identified several subclusters of luminal progenitor (LP, 1 to 5), hormone sensing (HS, 1 to 4), and basal (BSL, 1 and 2) cells. OA greatly affected the proportion of many cell subtypes. Notably, within the epithelial compartment, the proportion of BSL1, HS1, and LP3 increased by OA and the percentage of HS2, LP1, and LP4 decreased. In addition, the proportion of endothelial cells (EC) angiogenic tip cells decreased and the proportion of EC venous increased upon OA. The increase in proportions following OA exposure occurred in each of the donors to a similar extent (fig. S9).

### OA modulates gene expression in tissue-derived breast microstructures

**Fig. 5.**
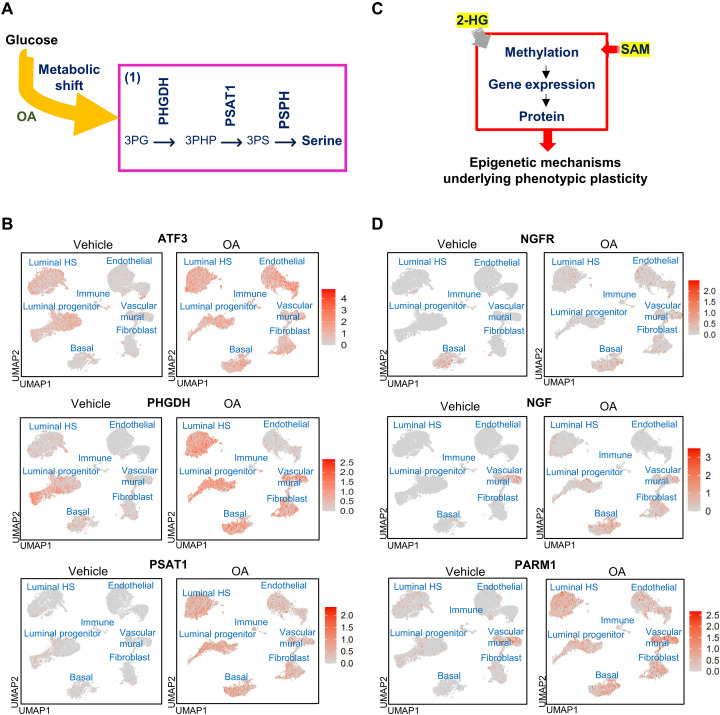
Feature plots of vehicle- and OA-treated cells from tissue-derived breast microstructures. (**A**) Metabolism of OA results in a metabolic shift toward the de novo serine pathway. (**B**) Feature plot of cells that are labeled according to *ATF3*, *PHGDH*, and *PSAT1* de novo serine-related genes. (**C**) Increases in 2-HG and SAM lead to epigenomic reprogramming. (**D**) Feature plot of cells that are labeled according to neural-related gene *NGFR*, *NGF*, and *PARM1* transcription, which are genes controlled by H3K4me3.

**Fig. 6.**
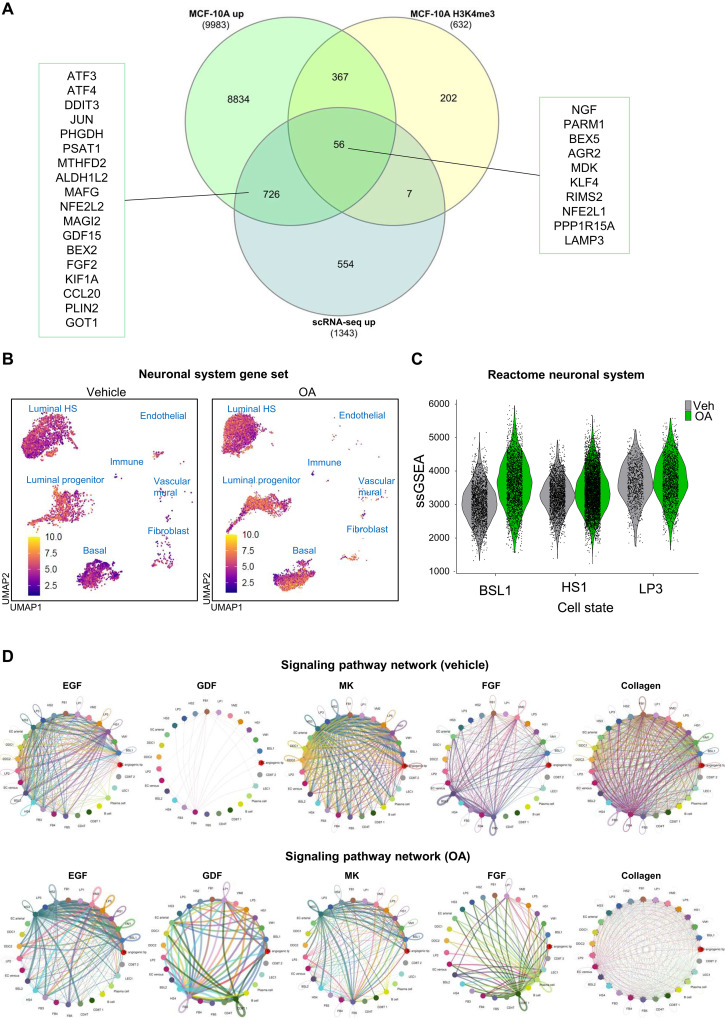
scRNA-seq analysis. (**A**) Venn diagram depicting genes up-regulated by OA in MCF-10A, genes with increased H3K4me3 peaks upon OA in MCF-10A, and up-regulated genes by OA in breast microstructures measured by scRNA-seq. (**B**) Feature plot of cells that are labeled according to neuronal system gene set. (**C**) Violin plot shows ssGSEA score of Reactome neuronal system in BSL1, HS1, and LP3 subtypes. (**D**) Circle plot of a selection of intercellular communication signaling network in OA and vehicle.

OA modulated gene expression within each subcluster. Figure 5 displays the previously identified de novo serine genes (*ATF3*, *PHGDH*, *PSAT1)* and neural-related genes (*NGF*, *NGFR*, *PARM1*). About 58% of OA-induced genes in breast microstructures overlapped with OA induced genes in MCF-10A cells (*14*), and about 4% of them had H3K4me3 peaks (Fig. 6A). The expression of many genes within each subcluster was modulated by OA. Notably, in BSL1, LP3, and HS1, OA induced the expression of genes involved in lipid droplets; epidermal growth factor receptor (EGFR) signaling; metabolism of serine, glycine, and folate; as well as endoplasmic reticulum stress, oxidative stress, and neural- and cancer-related genes, while lineage markers were repressed (table S2). Reactome pathway analysis revealed an up-regulation of terms related to the neuronal system (fig. S10). Neuronal system gene set was enriched in BSL1, LP3, and HS1 upon OA exposure (Fig. 6, B and C). Additional up-regulated terms were signal transduction, DNA repair, and metabolism, and down-regulated terms were related to cell-cell communication and extracellular matrix organization (table S3).

OA modulates gene expression within each cell subtype, leading to the down-regulation of lineage markers, notably in BSL1 and LP3. Therefore, we wondered whether OA induced changes in epithelial cell state. To answer this question, we remapped the cell populations using epithelial cell state markers provided by Kumar *et al.* (*23*) (fig. S11). Kumar and colleagues have identified three epithelial compartments: the luminal hormone–responsive (lumHR), luminal secretory (lumSec), also known as LP, and basal-myoepithelial (basal), and within each compartment, they have described several cell states. Figure S11 shows the proportion of cell states in vehicle and OA. Upon OA treatment, basal cells increased from 10% to 11%. Changes were observed in the lumHR and lumSec compartments. Within the lumHR compartment, most cells are in the lumHR-active state in the vehicle condition, while upon OA the proportion of lumHR-active decreased (from 13% to 6%); the proportion of lumHR-SCGB slightly increased (from 2% to 3%); and the proportion of lumHR-major expanded from about 4% to 20%. Within the lumSec compartment, the main OA-induced changes were observed in lumSec-basal (decreased from 19% to 8%), lumSec-prol (decreased from 5% to 0%), and lumSec-HLA (augmented from 1% to about 9%). This analysis shows that OA changed cell subtype proportions and clearly modulated gene expression within each cell compartment, leading to a change in cell state.

### Cell-cell communication landscape changes upon OA exposure

We used CellChat to explore the effect of OA in cell-cell communications. Enriched ligand-receptor pairs with up-regulated ligands in OA were different from those in vehicle. Among the signaling pathways that were highly active in OA were the growth factor midikine (MK), which is implicated in neurite outgrowth (*24*), epidermal growth factor (EGF), growth differentiation factor (GDF), and fibroblast growth factor (FGF). Collagen pathway was among the strongest contributors in cell-cell communication in vehicle (Fig. 6D). These results show that the overall interaction between cell subtypes changed upon exposure to OA, suggesting an increase of secreted signaling, a decrease of extracellular matrix–cell interactions, and a decrease of cell-cell adhesions.

### Compass algorithm predicts metabolic systems affected by OA in BSL1, LP3, and HS1 cell populations

**Fig. 7.**
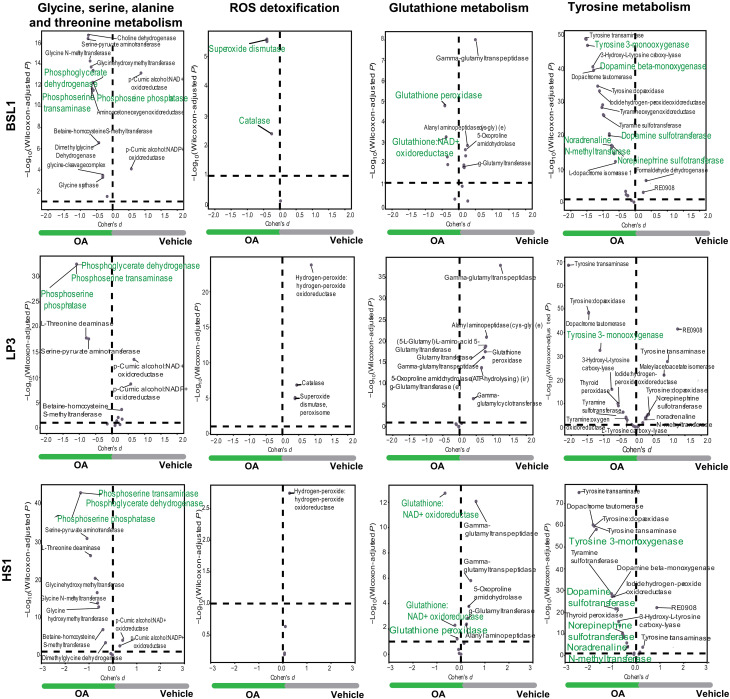
Metabolic systems affected by OA. Specific reactions in BSL1 (**top**), LP3 (**middle**), and HS1 (**bottom**) of glycine, serine, alanine, and threonine metabolism (reactions of the de novo serine pathway in green); ROS detoxification (catalase in green); glutathione metabolism (glutathione in green); and tyrosine metabolism (neurotransmitter-related reactions in green). Complete list of reactions in tables S8 to S10.

We performed metabolic flux analysis by using Compass to analyze our scRNA-seq data. Compass predicted differentially active metabolic reactions between OA and vehicle treatment in BSL1, LP3, and HS1 cells. This analysis showed that OA affects differently these three epithelial compartments, while greater OA effects are observed in BSL1 and HS1, and lesser OA effects are observed in LP3. Compass predicted increased dependence of BSL1, LP3, and HS1 cells on glycine, serine, alanine, and threonine metabolism upon OA exposure (Fig. 7); notably, key enzymes in serine metabolism such as PHGDH, phosphoserine aminotransferase 1 (PSAT1), and phosphoserine phosphatase (PSPH) were enhanced by OA in these cell subtypes. Compass highlighted differences in ROS detoxification and GSH metabolism among BSL1, LP3, and HS1 (Fig. 7). Catalase and superoxide dismutase reactions were enhanced by OA in BSL1 (Fig. 7). In BSL1 and HS1 cells, GSH metabolism pathway reactions were increased in vehicle and OA, but notably, GSH peroxidase (mitochondria) and GSH NAD^+^ oxidoreductase were increased only in OA (Fig. 7). In LP3 cells, GSH metabolism pathway was enhanced in vehicle (Fig. 7). The algorithm also predicted increased metabolic activity in tyrosine metabolism in all three cell subtypes (Fig. 7). Remarkably, metabolic reactions that result in the synthesis of neurotransmitters were enhanced in BSL1, LP3, and HS1 (Fig. 7). For example, dopamine β-monooxygenase and tyrosine 3-monooxygenase are in all three subtypes: dopamine sulfotransferase, norepinephrine sulfotransferase, and noradrenaline N-methyltransferase in BSL1 and HS1 (Fig. 7). This analysis confirmed the up-regulation of several metabolic reactions by OA; notably identified were the reactions catalyzed in the serine pathway in BSL1, LP3, and HS1, and increased ROS detoxification and GSH metabolism in BSL1, and OA led to increased flux in the reactions responsible for the synthesis of neurotransmitters in all three subtypes.

## DISCUSSION

**Fig. 8.**
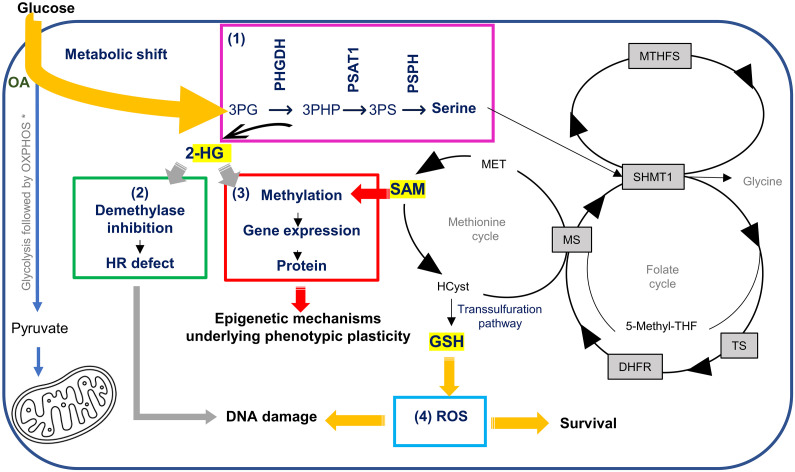
OA induced shift of metabolism away from glycolysis to the SOG/methionine cycle. (1) Metabolic shift leads to (2) increased 2-HG, which impairs DNA repair and contribute to epigenetic fostered plasticity; (3) increased SAM, which is responsible for epigenetic induced plasticity; and (4) increased glutathione (GSH) to counter the ROS and thus enabling survival of a subset of cells and contributing to DNA damage. SHMT1, serine hydroxymethyltransferase 1; TS, thymidylate synthase; DHF, dihydrofolic acid; DHFR, dihydrofolate reductase; MS, methionine synthase; MTHFS, methenyltetrahydrofolate synthetase; THF, tetrahydrofolate; MET, methionine; HCyst, homocysteine. Sections of this picture are shown in the previous figures.

Using in vitro and ex vivo models to study how a paradigmatic medium-chain fatty acid reprograms metabolism of nontransformed mammary cells and breast tissue, we demonstrate that OA in the presence of glucose, glutamine, and medium designed to mimic human plasma produces a metabolic shift from glycolysis to the SOG and methionine pathways that drives the production of SAM, GSH, and 2-HG (Fig. 8). This study represents a notable advancement beyond our previous work (*12*–*14*), providing valuable insights of the impact of OA exposure on metabolism, epigenomic reprogramming, and oxidative stress in nontransformed breast epithelial cells and tissue-derived breast microstructures. Many of the insights developed in our earlier studies were inferred from RNA sequencing. Here, we specifically measured the relative concentrations of various metabolites and assayed their downstream effects, e.g., methylation and mitigation of oxidative stress. Increased SAM provides an explanation as to how OA affects the changes in histone posttranslational modifications (PTMs) and gene expression we observed earlier (*14*). Flux through almost all reactions of the methionine cycle is significantly increased by OA (*14*); the status of the methionine component of one-carbon metabolism is sensed by histones to determine the levels of methylation on critical residues that mediate cellular epigenetic status (*25*). We have shown that OA produces significant increases in the methylation of various histones and that the increase in H3K4me3 leads to profound gene expression and phenotypic changes. 2-HG, which inhibits 2-oxoglutarate–dependent dioxygenases, thereby inhibits histone demethylases “locking in” the SAM-driven histone methylation changes. Finally, increased GSH may provide a survival advantage by mitigating oxidative stress in certain cell types, e.g., basal cells, within the mammary gland.

A key finding was the pivotal role played by PHGDH, which catalyzes the first and rate-limiting reaction of the de novo serine pathway. The association of *PHGDH* overexpression and ERneg BC was first observed over a decade ago. Possemato and colleagues developed a method using negative-selection RNA interference (RNAi) to identify metabolic genes required for in vivo tumorigenesis (*26*). Among the genes identified was PHGDH, which had the most significantly elevated expression in ERneg BC. A SOG pathway gene signature developed by Tedeschi and colleagues is significantly correlated with ERneg status [Pearson Correlation Coefficient (PCC) = 0.46, *P* = 1 × 10^−6^] (*27*). Locasale and colleagues correlated high PHGDH expression [immunohistochemistry (IHC) score > 1] with both triple-negative and basal subtypes (*28*). They also observed a phenotype using a tetracycline-inducible lentivirus vector that they developed to drive *PHGDH* expression in MCF-10A cells. In an in vitro acinus assay, this increased *PHGDH* expression resulted in disorganized acinar structures lacking lumen. The PHGDH-overexpressing cells within these structures lost apical polarity (*28*). PHGDH protein levels are elevated in 70% of ERneg BCs (*26*), which cannot be explained by gene amplification alone, as *PHGDH* amplification is observed in approximately 6% of all cases (*29*). This suggests that there are mechanisms other than gene amplification that contribute to *PHGDH* dysregulation (*26*). One of those mechanisms may be the fatty acid–induced metabolic shift toward the SOG and methionine pathways that we have identified. The transcription of the serine metabolic pathway enzymes is regulated by the transcription factors ATF4 and ATF3 (*30*, *31*), both induced by endoplasmic stress; one of the causes of endoplasmic stress is oxidative stress, which we have demonstrated in the ROS studies (Fig. 3, B and C). *ATF3* expression is increased in almost all cell subtypes in the scRNA-seq study, as are *PHGDH* and *PSAT1* (Fig. 6A). *PHGDH* and *PSAT1* are up-regulated in ERneg BC, and both have important roles in cancer development (*28*, *30*). The metabolic flux analysis of BSL1, LP3, and HS1 subtypes revealed that flux through the three enzymes of the de novo serine pathway—PHGDH, PSAT1, and PSPH—had among the greatest increases in flux secondary to OA exposure (Fig. 7). PHGDH also produces the oncometabolite 2-HG from αKG in the thermodynamically favored reverse direction (*15*). The appearance of DNA breaks as revealed by the comet assay is likely consequent to the increase of ROS. We considered whether the increased 2-HG may contribute to this phenomenon (*32*). Sulkowski and collaborators showed that the overproduction of 2-HG as a consequence of mutations in isocitrate dehydrogenase 1 and 2 impairs DNA repair. 2-HG inhibits the αKG-dependent dioxygenases such as KDM 4A/B. Their catalytic activity is required for HR repair; inhibition results in metabolic “BRCAness” (*21*). Under our assay conditions, we did not observe inhibition of HR. However, there may be intracellular conditions that produce 2-HG via PHGDH at a concentration high enough to inhibit HR.

We have been intrigued with the hints of neural differentiation, which first became evident from the data in our earlier paper (*14*). The increased production of SAM, the universal methyl donor, and 2-HG, a demethylase inhibitor (the involvement of 2-HG in demethylation and its impact on creating a highly plastic chromatin landscape has been demonstrated in BC) (*33*), leads to epigenetic, i.e., H3K4 trimethylation, fostered phenotypic plasticity. Hanahan has proposed that phenotypic plasticity is a discrete hallmark of cancer capability, and that nonmutational epigenetic reprogramming is a distinctive enabling characteristic that facilitates the acquisition of hallmark capabilities (*34*). This plasticity leads to reprogramming and/or differentiation to cells that express neural-, EMT-, stem cell–, and BC-related genes. Network analysis of pathways associated with H3K4me3 peaks upon OA exposure revealed several neural-related pathways. Pathways that related to neural development were also overrepresented in breast microstructures upon OA, e.g., signaling by NTRKS (neurotrophic tyrosine receptor kinase) and nerve growth factor (NGF) stimulated transcription that have been associated with ERneg BC (*35*), stem cell self-renewal, and plasticity (*36*); MK signaling, which is involved in neurogenesis and cancer progression (*24*); and synthesis of neurotransmitters, which can be produced by nonneuronal cells including BC cells (*37*). Of note, BC is one of the malignancies in which a neural differentiation phenotype has been observed, specifically in triple-negative BC, a subtype of ERneg BC (*38*). This specific differentiation, i.e. neural, may be a key feature of tumorigenesis, which is hypothesized to represent the culmination of a process of gradual loss of a cell’s original identity and gain of the properties of neural progenitor cells (*39*–*41*). A recently added “Hallmark of Cancer” is the intersection with neurobiology (*42*). In discussing this hallmark, Hanahan and Monje reflect on the fact that the expression of neuronal signaling and regulatory circuits are observed in cancer cells of multiple origins, not just ones with ontological relationships to neurons (*42*). They also note that cancer cells can also exhibit distinctly neuronal structural features, such as the extension of long, neurite-like processes that facilitate cell-to-cell communication in the tumor microenvironment (*42*). Axonogenesis and neurogenesis are observed in premalignant lesions, for example, in the initial stages of prostatic intraepithelial neoplasia (PIN) (*43*); interestingly, the data presented here demonstrates that this occurs in normal tissue and is consequent to fatty acid exposure.

Live monitoring of ROS-induced redox changes revealed a significant ROS increase shortly after OA exposure. ROS are controlled by antioxidant defenses. This probably favors/enables the survival of specific cell subtypes that can mitigate ROS. Metabolic flux analysis of our scRNA-seq data revealed that GSH metabolism and ROS detoxification was increased only in BSL1. This may not be surprising as previous scRNA-seq of primary human breast epithelial cells has revealed that the three subtypes of breast epithelial cells—luminal mature (or HS), LPs, and basal—have distinct metabolic properties (*44*). Earlier studies have demonstrated that basal cells maintain low levels of ROS primarily by engaging a GSH-dependent antioxidant mechanism, while LPs have a robust antioxidant defense as they can mitigate higher levels of ROS relying on multiple GSH-independent antioxidants (*45*); this may be reflected in the increased survival of LP3 cells despite the down-regulation of GSH peroxidase. LPs are posited to be the cells of origin of basal-like BCs (an ERneg BC subtype), and it has been shown that under nonphysiological conditions they can acquire stem-like features, suggesting a remarkable degree of plasticity. It is possible that a subset of LPs that can survive and alternate their phenotype are ripe for malignant transformation; however, further experiments would need to be performed to explore this possibility.

Our data represent a compendium of in vitro and ex vivo metabolic, epigenomic, and molecular data on the effects of OA, in the context of human plasma-like medium (HPLM). A detailed discussion of the choice of OA and its concentration is provided in the Supplementary Methods. Medium-chain fatty acids can be esterified in triacylglycerides. OA in adipose cells becomes saturated at ~10% of total fatty acids. This represents approximately 20% of the total acyl chains stored in adipose cells, an amount that would be predicted to have substantial effects on cellular metabolism (*46*). OA freely diffuses across cell membranes due to its small size and lipophilic nature, enabling the delivery of a reliable concentration and reproducibility across experiments. Previously, we assayed gene expression changes in MCF-10A cells consequent to exposure to the long-chain, polyunsaturated fatty acid, linoleic acid (LA). The number of statistically significant changes in gene expression was considerably fewer in the LA-treated cells when compared to the OA-treated cells (*14*). This difference has also been observed in adult rat cardiac myocytes (*47*). Entry of long-chain fatty acids into the mitochondria is strictly regulated by carnitine palmitoyl transferase 1 (CPT1), whereas the medium-chain fatty acids freely diffuse into the mitochondria, which may be a substantial vulnerability given the profound changes in gene expression we have observed. The use of only one fatty acid is a limitation of our study. We recognize that additional fatty acids should continue to be explored; however, the current paucity of information regarding the lipid content of the normal breast limits our ability to make an informed selection. Mammary cells have access to lipids from serum or the adjacent adipocytes of the breast adipose tissue. From coculture studies of BC cell lines and adipocytes, it has been shown that adipocyte-derived fatty acids drive proliferation and migration (*48*). Lipid uptake, for example, that mediated by fatty acid translocase/CD36 and fatty acid binding protein 4 (FAB4), facilitates fatty acid transport into the mammary epithelial cells and has been shown to play critical roles in proliferation, migration, and metastasis of BC cells (*49*, *50*). Although the relationship between dietary fat intake and BC risk remains controversial, breast adipose tissue measurement in pre- and postmenopausal women and breast lipid composition imaging evaluation in postmenopausal women revealed an association between saturated fatty acids and BC (*51*, *52*). In addition, a high saturated fat diet during puberty or adulthood increased the incidence of mammary tumors in mouse models (*53*).

We have demonstrated that in vitro and ex vivo exposure to fatty acids can produce profound consequences on mammary cells/tissue. The effects intersect many of the hallmarks of cancer including deregulating cellular metabolism, unlocking phenotypic plasticity (*34*), and a recently added hallmark: the intersection with neurobiology (*42*). Our experimental results suggest at least two potential mechanisms of tumorigenesis; the increased ROS in the nucleus (Fig. 3, B and C) suggests the possibility of oxidation of nucleotides within the DNA, deoxyguanine the likely target. Mutational signature 18, which is thought to occur secondary to DNA damage from ROS, is one of the signatures observed in BC (https://cancer.sanger.ac.uk/signatures/sbs/sbs18/). Second, we have shown that 2-HG concentrations are increased by OA. Although the concentrations of 2-HG produced under the experimental conditions that we used were inadequate to affect HR, 900 μM 2-HG, which we used as the positive control, clearly inhibited HR. We have only begun to probe intracellular conditions that produce 2-HG, and it is likely that some conditions will result in concentrations high enough to inhibit HR. For example, the reaction catalyzed by PHGDH is driven toward 3-phosphohydroxypyruvate (3-PHP) due to 3-PHP’s consumption by downstream pathway steps (*15*); if this does not occur, catalysis will be toward the production of 2-HG.

The limitations of our study include using a single saturated fatty acid (OA) in the experiments reported in this study. Additionally, microstructures from only two donors were assayed by scRNA-seq. To date, we have only examined two of the histone methylation marks statistically significantly increased by OA. CBR-5884 has been tested at two doses in MCF-10A cells (*54*). We used a single concentration and the higher of the two: 30 μM.

Future directions will include returning from “bench to bedside” to access our CUB tissue collection to determine whether the histone PTM and changes in gene expression we have observed in the cell lines and breast tissue microstructures are present in the CUBs. We will assay as a function of the ER status of the index lesion and will also assay healthy breast tissue. To address the limitation of assaying only a single fatty acid at a time, we will perform untargeted lipidome profiling of the same CUBs and healthy tissue. This has the potential to inform whether the lipidome differs as a function of ER status and, if so, provide a list of candidate lipids to test. We recognize the importance of developing in vivo data from animal models with the goal of elucidating an unambiguous connection of the lipids we identify in the CUBs to ERneg BC oncogenesis.

## MATERIALS AND METHODS

### Cell culture

MCF-10A cell line was obtained from the American Type Culture Collection (ATCC; #CRL-10317) and cultured in mammary epithelial cell growth basal medium (MEBM) with single quots supplements and growth factors (Lonza, #CC-4136). Alternatively, cells were cultured in HPLM (Gibco, #A4899101) supplemented with H14 medium additives (*55*). Cells were regularly screened for the absence of mycoplasma contamination with the ATCC Universal Mycoplasma Detection Kit (ATCC, #301012K).

### Glucose ^13^C tracing

For isotope tracing experiments, cells were seeded in biological triplicate (∼80% confluent) in HPLM plus H14 supplements and treated with 5 mM of the medium-chain fatty acid OA (Sigma-Aldrich, #C5038) dissolved in phosphate-buffered saline (PBS) or vehicle (PBS) for 20 to 23 hours. Cells were washed once with RPMI 1640 medium without glucose (Gibco, #11879020) and then incubated in RPMI 1640 medium without glucose containing d-glucose-^13^C_6_ (900.8 mg/liter; Sigma-Aldrich, #389374), supplemented with H14 medium additives (*55*), 700 μM alanine (Sigma-Aldrich, #A7627), 150 μM cysteine (Sigma-Aldrich, #C1276), 40 μM pyruvate (Gibco, #11360070), and either vehicle or 5 mM OA. After 1, 2, or 4 hours, metabolites were extracted as described previously (*56*). Briefly, cells were rinsed twice with 5 ml of ice-cold saline solution, 1 ml of 80% methanol (Sigma-Aldrich, #34860) cooled to −80°C was added, and cells were scraped on dry ice. Cell lysates were incubated at −80°C for 5 min and vortexed for 1 min at room temperature; after repeating these steps twice, cell lysates were incubated at −20°C overnight. Lysates were then vortexed for 30 s, and insoluble material was pelleted by centrifugation at 20,000*g* for 15 min at 4°C. Supernatant was collected for high-performance liquid chromatography and high-resolution mass spectrometry and tandem mass spectrometry (HPLC-MS/MS) analysis. Data were analyzed at 4 hours after ^13^C-label addition.

### Analysis of ^13^C tracing data

We constructed a metabolic network model that covers most fluxes in glycolysis, TCA cycle, pentose phosphate pathway, one-carbon metabolism pathway, and amino acid synthesis pathway. To reduce random errors in batch preparation and measurement, mass isotopomer distributions (MIDs) of metabolites in all biological repeats are averaged. These average MID data are fitted with the following procedure: MIDs of all target metabolites are predicted by averaging the MID of the precursors, weighted by the corresponding generating fluxes with the presumed value

$${\tilde{M}}_{i}=\frac{\sum_{\forall j}v_{\mathrm{ji}}M_{\mathrm{ji}}}{\sum_{\forall j}v_{\mathrm{ji}}}$$

${\tilde{M}}_{i}$: predicted MID vector of metabolite *i*; *M_ji_*: MID of metabolite i produced from a substrate *j*; *v_ji_*: the flux from *j* to *i*.

If *M_ji_* is still unknown, it can be deduced by the same procedure, until MIDs of all precursors are known. Then, the difference between the predicted and experimental MIDs of target metabolites was evaluated by Kullback-Leibler divergence

$$L_{i}=D_{\text{KL}}\left({\tilde{M}}_{i}∥M_{i}\right)=\sum_{j}M_{i,j}\text{log}\frac{{\tilde{M}}_{i,j}}{M_{i,j}}$$

*L_i_*: difference of target metabolite *i*. *M_i,j_*, ${\tilde{M}}_{i,j}$: element *j* in vector *M_i_* and ${\tilde{M}}_{i}$.

The sum of *L_i_* for all target metabolites was regarded as the total difference $L_{\text{total}}$ to minimize by adjusting flux vector $v=\left\{v_{i}\right\}$ that includes all fluxes. Therefore, an optimization problem was defined as

$$\underset{v}{\text{min}}L_{\text{total}}\left(v\right),s.t.A\cdot v^{T}=b,0\leq v_{\text{min}}\leq v\leq v_{\text{max}}$$

$A\cdot v^{T}=b:$ flux balance requirement and other equality constraints; $v_{\text{min}}$, $v_{\text{max}}$: lower and upper bounds for composite vector v.

The solution of this optimization problem $v^{*}$ gives a combination of all fluxes in the network model that fits MID data. The method used to solve this optimization problem refers to previous publication (*57*). For better precision, accuracy, and robustness, the optimization is repeated 10,000 times and the 50 solutions with minimal final $L_{\text{total}}$ are selected to be the final solution set.

### Targeted metabolomics

To determine the relative abundances of intracellular metabolites over time, MCF-10A cells seeded in biological triplicate in HPLM plus H14 supplements were treated with vehicle [PBS or PBS plus dimethyl sulfoxide (DMSO)], 5 mM OA, and 5 mM OA plus 30 μM of the PHGDH inhibitor CBR-5884 (Sigma-Aldrich, #SML1656). Metabolites were prepared as described in the glucose C13-glucose tracing experiment and collected for LC-MS analysis. The targeted metabolites serine, SAM, glycine, αKG, sarcosine, glycine betaine, methylglyoxal, 2-HG, GSH, phosphoribosyl diphosphate (PRPP), betaine aldehyde, and NADH were measured after 0 min, 5 min, 15 min, 1 hour, 2 hours, and 4 hours of exposure.

### Quantification of d-2-HG and l-2-HG

To quantify 2-HG enantiomers d-2-HG and l-2-HG, MCF-10A cells (biological triplicate) seeded in HPLM plus H14 supplements were exposed with vehicle (PBS) and 5 mM OA for 30 min. In addition, MCF-10A cells were transfected with the human ON-TARGET plus small interfering RNA (siRNA) library targeting PHGDH (Dharmacon) or nontarget control (Dharmacon) using the Neon Transfection System (Invitrogen). Then, 96 hours after transcription, cells were exposed to vehicle (PBS) and 5 mM OA for 30 min. Metabolites were then prepared as described in the glucose C13-glucose tracing experiment. l-2-HG and d-2-HG derivatization was performed as previously described (*58*). The amount of l-2-HG and d-2-HG in extracts was quantified by using a calibration curve and normalized to the number of cells.

### Analysis of mitochondrial and nuclear ROS using in vivo reporters

ROS-induced redox changes were monitored using ORP1-roGFP2–based sensors in MCF-10A cells. NLS-roGFP2-Orp1 and mito-roGFP2-Orp1 (puromycin as selection mark) plasmids were obtained from cloning the c-myc sequence or a mitochondrial targeting sequence (MTS) sequence, respectively, into the pEIGW roGFP2-ORP1 vector (Addgene, #64993). For lentivirus production, human embryonic kidney (HEK) 293T/17 cells (ATCC, #CRL-11268) in phenol red–free Opti-MEM (Gibco) were cotransfected with a construct of interest and packaging plasmids Gag-Pol 8.91 (Addgene, #187441) and VSV-G (Addgene, #8454) using Lipofectamine 3000 (Thermo Fisher Scientific). Cells were incubated at 37°C, and after 24 and 48 hours, lentivirus-rich medium was collected and centrifuged (500*g*, 5 min). Supernatant was collected, filtered (0.45 μm), and used for transduction. Transduced MCF-10 cells were cultured in the appropriate medium for 48 hours and selected with antibiotics. Cells were imaged in Lionheart FX (Biotek). roGFP2-Orp1 was excited sequentially at 405 and 488 nm, and the emission was recorded at 525 nm. The generated images were analyzed using FIJI ImageJ (1.53c). Each channel was converted to 8-bit, and median (radius = 2 pixels) and Gaussian blur (radius = 2 pixels) were applied. The background was subtracted, and a threshold was set to avoid artifacts. Only the nuclear or mitochondrial area was considered for the analysis. Oxidation levels were then determined on a pixel-by-pixel basis by dividing the signal emitted for each channel (405 nm/488 nm) using Imaging Expressing Parser. Final ratiometric values were obtained using Analyzing particles (size: 10 μm^2^ to infinity; circularity: 0.50 to 1.00). Obtained values were plotted in GraphPad Prism 10, and a heatmap (Lookup table: Fire) was created for visualization.

### Comet assay

The alkaline comet assay (Abcam, #ab238544) for assessing DNA damage in MCF-10A cells was performed according to the manufacturer’s protocol. Slides were viewed with a Lionheart FX (Biotek) using a fluorescein isothiocyanate (FITC) filter. Images were analyzed by the ImageJ software (National Institutes of Health) using OpenComet v1.3.1 (*59*).

### CUT&RUN sequencing

For CUT&RUN, cells were seeded in biological triplicate (∼80% confluent) in complete MEBM medium and exposed to 5 mM OA or vehicle (PBS) for 24 hours. About 900,000 cells were processed using the CUTANA kit (Epicypher, #14-1048). Briefly, MCF-10A cells were detached by exposure to 0.25% trypsin-EDTA (Gibco, #15400054) for 1 min, and then cells were scrapped off the culture plates and centrifuged at 600*g* for 3 min. Pellets were washed twice with CUT&RUN wash buffer and then mixed with concanavalin A (ConA) beads. Following a 10-min incubation on a magnet rack at room temperature, the supernatant was discarded. Samples were resuspended in antibody buffer, and antibodies were added to the beads along with the bound cells as follows: 1:100 H3K4me3 antibody (Epicypher, #13-0041), 1:50 H3K27me3 antibody (Cell Signaling, #9733), and 1:100 rabbit immunoglobulin G (IgG) negative control antibody (Epicypher, #13-0042) and incubated overnight at 4°C. Then, the supernatant was removed and beads were washed twice with cell permeabilization buffer. Next, Protein A/G-micrococcal nuclease (pAG-MNase) was added to the sample for 10 min at room temperature followed by two washes with cell permeabilization buffer. Then, CaCl_2_ was added to a final concentration of 2 mM to activate MNase and initiate chromatin cleavage. Samples were incubated for 2 hours at 4°C. Following incubation, STOP buffer was added and cells were incubated at 37°C for 10 min to release chromatin fragments. DNA was purified using the CUTANA DNA purification kit. The purified DNA was quantified using Qubit (Invitrogen) as per the manufacturer’s instructions. Library preparation was performed using the NEBNext Ultra II Library Prep Kit for Illumina (New England BioLabs, #E7645S) per the manufacturer’s instructions with minor modifications. Following adapter ligation, DNA cleanup was performed using 1.0× AMPure XP beads (Beckman Coulter Inc., #A63880). PCR was performed using unique dual index primer pairs (NEBNext Multiplex Oligos for Illumina from New England BioLabs, #E6440S) according to the following parameters: 45 s at 98°C to activate hot-start Q5 polymerase, followed by 15 s at 98°C, 10 s at 65°C for a total of 13 cycles, and finally 1 min at 72°C for final extension. DNA cleanup was performed using 1.0× AMPure beads (Beckman Coulter Inc., #A63881), and the DNA was eluted in Tris-EDTA (TE) buffer. DNA quantification was performed using Qubit (Invitrogen), and fragment sizes of individual libraries were analyzed using the High Sensitivity DNA Kit (Bioanalyzer). Libraries were pooled to a final concentration of 23 nM and sequenced on NovaSeq 6000 (Illumina), 100–base pair paired-end reads.

### CUT&RUN data analysis

MACS2 v2.2.7.1 was used to call H3K4me3 narrow peaks, and SICER2 was used to call H3K27me3 broad peaks. The differential binding regions were identified using DiffBind v3.9. For H3K4me3, default summit parameter was used. For H3K27me3, summits = 1000 was used. ChIPseeker 1.8.6 was used to annotate the differential peaks identified by DiffBind. HOMER v4.11 was used to scan for the enrichment of motifs using default parameters. CUT&RUN data were compared to published bulk RNA sequencing data (*14*). The Enrichr web was used to perform pathways analysis (https://maayanlab.cloud/Enrichr/, accessed on 14 September 2023) (*17*).

### Culture in poly-d-lysine/laminin plates

MCF-10A, MCF10ADCIS.com cells (RRID:CVCL_5552), and cells derived from breast microstructures were plated in poly-d-lysine/laminin (PDL/LM) chamber slides (Corning, #354595) treated with vehicle or 5 mM OA for 24 hours. Pictures were taken using an EVOS Digital Microscope.

### Mammary microstructure preparation

Tissues were collected from women admitted for reduction mammoplasty (noncarriers) or from prophylactic mastectomy specimens (BRCA1 mutation carriers) at Prentice Women’s Hospital of Northwestern Medicine recruited under an approved institutional review board protocol (NU15B07) by Northwestern’s Institutional Review Board. All participants provided written informed consent. Breast tissue to be processed was transferred into a sterile petri dish, chopped into small pieces using scissors, and then transferred to sterile 50-ml tubes containing collagenase (1 mg/ml) from *Clostridium histolyticum* (Sigma-Aldrich, #C0130) in Kaighn’s modification medium (Gibco, #21127022) supplemented with 0.5% BSA (Sigma-Aldrich, #SLCM0392) and antibiotic-antimycotic (Gibco, #15240062). A 0.22-μm filter was used to filter the medium containing collagenase. Falcon tubes were sealed with tape, and tissue was gently dissociated on a shaker at 100 rpm and 37°C overnight (16 hours). The next day, microstructures were collected and washed with PBS by centrifugation (114*g* for 5 min). Microstructures were resuspended in fresh HPLM medium (Gibco, #A4899101) supplemented with H14 medium additives plus antibiotic-antimycotic (Gibco, #15240096) and added to ultra-low attachment surface six-well plate (Corning, #CLS3471).

### Quantitative reverse transcription PCR

MCF-10A cells were exposed in biological triplicate to vehicle [PBS or PBS plus dimethyl sulfoxide (DMSO)], 5 mM OA, 5 mM OA plus 30 μM CBR-5884, or 5 mM OA plus 160 μM Piribedil for 24 hours. RNA was then isolated with Qiagen AllPrep DNA/RNA/Protein Mini Kit (Qiagen, #80004). Breast microstructures from 12 independent donors were exposed to vehicle (PBS) and 5 mM OA for 24 hours. In a separate experiment, breast microstructures from 12 independent donors were exposed to vehicle (PBS plus DMSO) 5 mM OA, or 5 mM OA plus 15 μM clofazimine (MedChemExpress, #HY-B1046) for 24 hours. After incubation, microstructures were washed with PBS and RNA was isolated with Qiagen AllPrep DNA/RNA/Protein Mini Kit (Qiagen, #80204). cDNA was synthesized using the SuperScript VILO cDNA synthesis kit (Thermo Fisher Scientific, #11755250). RT-qPCR was performed using Applied Biosystems QuantStudio 5 Real-Time PCR System (Thermo Fisher Scientific). PrimeTime qPCR probe assays (tables S4 and S5) and PrimeTime Gene Expression Master mix were purchased from Integrated DNA Technologies (IDT). For the fold change analysis, 2^−ΔΔ*C*T^ was calculated as described elsewhere (*60*). For this, gene of interest levels were normalized relative to the mean expression of the three control genes *UBB*, *RPLP0*, and *TBP* (Δ*C*_T_). The difference between treated and untreated gave the ΔΔ*C*_T_.

### Single-cell preparation

Breast microstructures from two donors were exposed to OA or vehicle (PBS) for 24 hours. Microstructures were then collected into a 50-ml conical tube by centrifugation (179*g* for 5 min) and washed with Hanks’ balanced salt solution (HBSS) without calcium and magnesium (Gibco, #14175095) containing 0.1% bovine serum albumin (BSA) (Ambion, #AM2616). After 5-min centrifugation (179*g* for 5 min), collected microstructures were incubated with prewarmed (37°C) TrypLE (Gibco, #12604013) for 15 min in a shaker at 100 rpm and 37°C. Every 5 min, the dissociation mixture was pipetted gently using wide-orifice p1000 tips. Prewarmed complete HPLM medium containing 0.1% BSA was added to the cell suspension and cells were collected by centrifugation (200*g* for 5 min). Cells were incubated with 2 ml of RBC lysis buffer (Invitrogen, #00433357) for 1 min at room temperature, prewarmed complete HPLM medium containing 0.1% BSA was added to the cell suspension, and cells were collected by centrifugation (200*g* for 5 min). Cells were resuspended in 1 ml of complete HPLM medium containing 5 mg of Dispase II (Sigma-Aldrich, #D4693) and 0.1 mg of deoxyribonuclease (DNase) I (STEMCELL Technologies, #07469) and incubated for 3 min in a shaker at 200 rpm and 37°C. After adding complete HPLM medium, cells were passed three times through an 18-gauge needle attached to a 10-ml syringe and filtered through a 40-μm cell strainer. Additional three washes with cold complete HPLM medium were performed (200*g* for 5 min at 4°C). Single cells were then suspended in HBSS containing 0.1% BSA.

### scRNA sequencing

For scRNA-seq, the cells were stained with trypan blue to count and determine cell viability. Cells were suspended in PBS/0.04%BSA. About 10,000 individual cells per sample were mixed with Master Mix and loaded onto a 10x Genomics Chromium Single Cell instrument according to the manufacturer’s instructions. All the procedures were done using Chromium Next GEM Single Cell 3′ Reagent kits v3.1, dual indexing, following the 10x Genomics user guide #CG000315. All four libraries passed the quality control (QC) and were sequenced into one S4 lane NovaSeq 6000 with configuration 28 × 10 × 10 × 150. About 2.8 billion clusters were processed to generate fastq files.

### Processing and annotation of scRNA-seq data

Raw sequencing data were processed using CellRanger version 7.0.1. Raw sequencing reads were aligned against the GRCh38 human genome with corresponding gene model from Ensembl database version 109. The digital expression matrix file containing UMIs (unique molecular identifiers) was also generated. scRNA-seq analysis was performed using Seurat version 4.3.0 and R version 4.2.2 (*61*). The following filtering criteria were used: nFeature_RNA > 2000 and nFeature_RNA < 10,000; % mitochondrial gene <20%; and % ribosomal genes <30%. DoubletFinder package version 2.0.3 was used to remove doublets. The NormalizeData function was used for normalization (*62*). The R package Harmony version 0.1.1 was used for batch correction and integration (*63*). Unsupervised clustering identified 25 clusters (resolution of 0.8). FindAllMarkers function in Seurat was used to identify differentially expressed genes in each cluster compared to the rest of the cells (*P* < 0.05 and log fold change ≥ 0.25). SingleR package (version 2.0.0) (*64*) was used for cell annotation according to the Reed *et al.* (*65*) and Kumar *et al.* (*23*) reference datasets.

### Single-cell pathway analysis

Pathway analysis was performed using SCPA (1.2.0) with msigdbr v7.5.1 Reactome pathways (*66*). Reactome pathways are provided in table S3. Single-cell pathway analysis plots for neuro-related terms were made. The escape R package (v1.99.1) with single-sample gene set enrichment analysis (ssGSEA) was used to map the Reactome neuronal system gene set.

### Cell-cell communication network analysis

Cell-cell interactions among cell subpopulations were investigated using R package CellChat version 1.6.1 (*67*). For every pair of cell types, cell-cell communication interaction was identified.

### Metabolic pathway inference

The Compass algorithm was used for in silico single-cell flux balance analysis (FBA) (*68*). Each cell subset (BSL1, LP3, HS1) was renormalized using the Seurat NormalizeData function with relative count “RC” normalization and a scale factor of 10,000. Compass was run using standard settings. FBA reaction penalties for each RECON2 metabolic subsystem were negative log transformed, and Wilcoxon rank-sum test was used to identify reaction subsystems predicted to be altered with/without OA treatment. Predicted changes in metabolic activity are represented by a Cohen’s *d* value (mean divided by the pooled SD).

## Supplementary Materials

### The PDF file includes:

Supplementary Methods Figs. S1 to S11 Legends for tables S1 to S10 References
