# Supplementary material for: A metabolic shift to the serine pathway induced by lipids fosters epigenetic reprogramming in nontransformed breast cells
Source: Sci Adv. 2025 Mar 21;11(12):eads9182. doi: 10.1126/sciadv.ads9182 (PMC11927636; doi:10.1126/sciadv.ads9182)
Supplement: Supplementary file 1 — Supplementary Methods Figs. S1 to S11 Legends for tables S1 to S10 References [file sciadv.ads9182_sm.pdf]

Science Advances
AAAS

Supplementary Materials for

A metabolic shift to the serine pathway induced by lipids fosters epigenetic reprogramming in nontransformed breast cells

Mariana Bustamante Eduardo *et al.*

Corresponding author: Susan E. Clare, susan.clare@northwestern.edu

Sci. Adv. **11**, eads9182 (2025)
DOI: 10.1126/sciadv.ads9182

The PDF file includes:

Supplementary Methods
Figs. S1 to S11
Legends for tables S1 to S10
References

Other Supplementary Material for this manuscript includes the following:

Tables S1 to S10

Supplementary methods

Selection of Octanoic acid (OA) concentration

We began these experiments using 5 mM OA and MCF-10A cells. OA, for the purposes of these experiments serves as the paradigmatic medium-chain fatty acid. 5mM was chosen as a starting concentration based upon McDonnell *et al.* (*19*). Our initial hypothesis was that lipids would alter histone acetylation rather than methylation in non-transformed breast epithelial cells as OA-mediated histone acetylation had been demonstrated in two malignant cell lines, MCF7 and MDA-MB-231, in McDonnell *et al.* (*19*). Therefore, histone acetylation was used as a readout to determine OA concentration. We observed H3K14 acetylation at both 2 mM and 5 mM OA but H3K9 acetylation only at 5 mM (14); therefore, we continued experiments at 5 mM. Subsequent analysis of histone acetylation and methylation utilizing the Epiproteomic Histone Modification Panel revealed that OA increased histone methylation more significantly than histone acetylation; this fact borne out by subsequent methylation and acetylation flux analysis (*14*).

Generation of PHGDH overexpressing MCF-10A cells

MCF-10A cells were transduced with lentiviral vectors prepared and used as previously described (*28*). In brief, MCF-10A cells transduced with the lentiviral vector pLvx-Tight-Puro encoding for PHGDH. Stably transduced cells were selected with 1 µg/ml puromycin (Gibco, # A1113803). PHGDH was induced with 2 µg/ml doxycycline (Sigma-Aldrich, # D9891).

ALDEFLUOR® assay

For ALDEFLUOR® assay MCF-10A cells seeded in HLPM plus H14 additives were exposed to 5mM OA or vehicle (PBS) for 24 hours in biological triplicates. The ALDEFLUOR® assay (Stemcell Technologies**,** #01700) was used according to the manufacturer's instructions. Briefly, 1 x 10^6 cells/mL cells were suspended in ALDEFLUOR assay buffer containing the ALDH substrate BODIPYaminoacetaldehyde and incubated at 37°C for 60 min. For each sample, cell aliquots were incubated with or without 50 mM diethylaminobenzaldehyde (DEAB), an ALDH-specific inhibitor. ALDH analysis was performed using a FACSymphony A5-Laser Analyzer.

Cell viability

MCF-10A cells were exposed in biological triplicate to vehicle (PBS plus DMSO), 5mM OA ± 1mM N-acetylcysteine (NAC, Sigma-Aldrich # A7250), 5mM OA plus 30µM CBR-5884 ± 1mM NAC, or 5 mM OA plus 15 µM Clofazimine ± 1mM NAC for 24 hrs. Cells were suspended in a suspension volume of 200 µl. A total of 10 ul was stained with 10 µl of 0.4% (v/v) trypan blue. A 10-µl sample was placed on a Bright-Line hemocytometer (Hausser Scientific, Horsham, PA, USA) and counted using × 40 magnification.

Luciferase-based host-cell reactivation reporter assays for homologous DNA repair

The HR luciferase reporter assay has been used to assess homologous recombination as a function of the restoration of luciferase expression as previously reported (*21*). The firefly luciferase gene was digested by I-SceI and confirmed by electrophoresis. A promoter-less luciferase donor DNA is located downstream as a template for homologous DNA repair. A total of 1 µg of the I-SceI digested luciferase reporter or the luciferase-

reporter control plus 50 ng Renilla luciferase vector were transfected into MCF-10A cells using the Neon Electroporation System 100 µl kit (Invitrogen, # MPK10025) with the following parameters: 500,000 cells, 1250V, 20 ms, 2 pulses. Luciferase-reporter assays were performed in 12-well plates. Twenty-four hours after transfection cells were exposed to vehicle, Octanoic acid (OA) or 900 µM 2-HG (Stemcell Technologies Inc., # 50-206-9334). Cell lysed 73 hours post-transfection using Passive Lysis Buffer, transferred to 96-well white flat-bottom plates and assayed for luciferase activity with the Dual-Luciferase Reporter Activity system (Promega, #E1910) using a Biotek Cytation 3 multiwell reader. All transfections were performed in triplicate. Luciferase measurement for each biological replicate was performed in three technical replicates. Firefly luciferase activity was normalized to Renilla luciferase activity for transfection efficiency. The % of reporter expression was calculated by diving the normalize value of each digested luciferase replicate by the mean of the normalize value of the three luciferase controls multiply by 100. Bars represent mean +/− SEM. Statistical analysis by t-test (n=3).

DNA methylation

DNA was isolated from MCF-10A cells exposed to vehicle (PBS or PBS plus DMSO), 5mM OA and 5mM OA plus 30µM CBR-5884 for 24 hours with Qiagen AllPrep DNA/RNA/Protein Mini Kit (Qiagen # 80004). DNA (250–500 ng) was treated with sodium bisulphite using the EZ DNA methylation kit (Zymo Research, # D5001) and Genome-wide DNA methylation profiling was performed using the Illumina Infinium Methylation EPIC Beadchip array. Raw IDAT files were processed using the GenomeStudio software V2011.1. The infinium-methylationepic-v-1-0-b5-manifest-file.bpm manifest was used to process EPIC data. All downstream analysis was conducted using SeSAMe (*69*).

Supplementary Figures

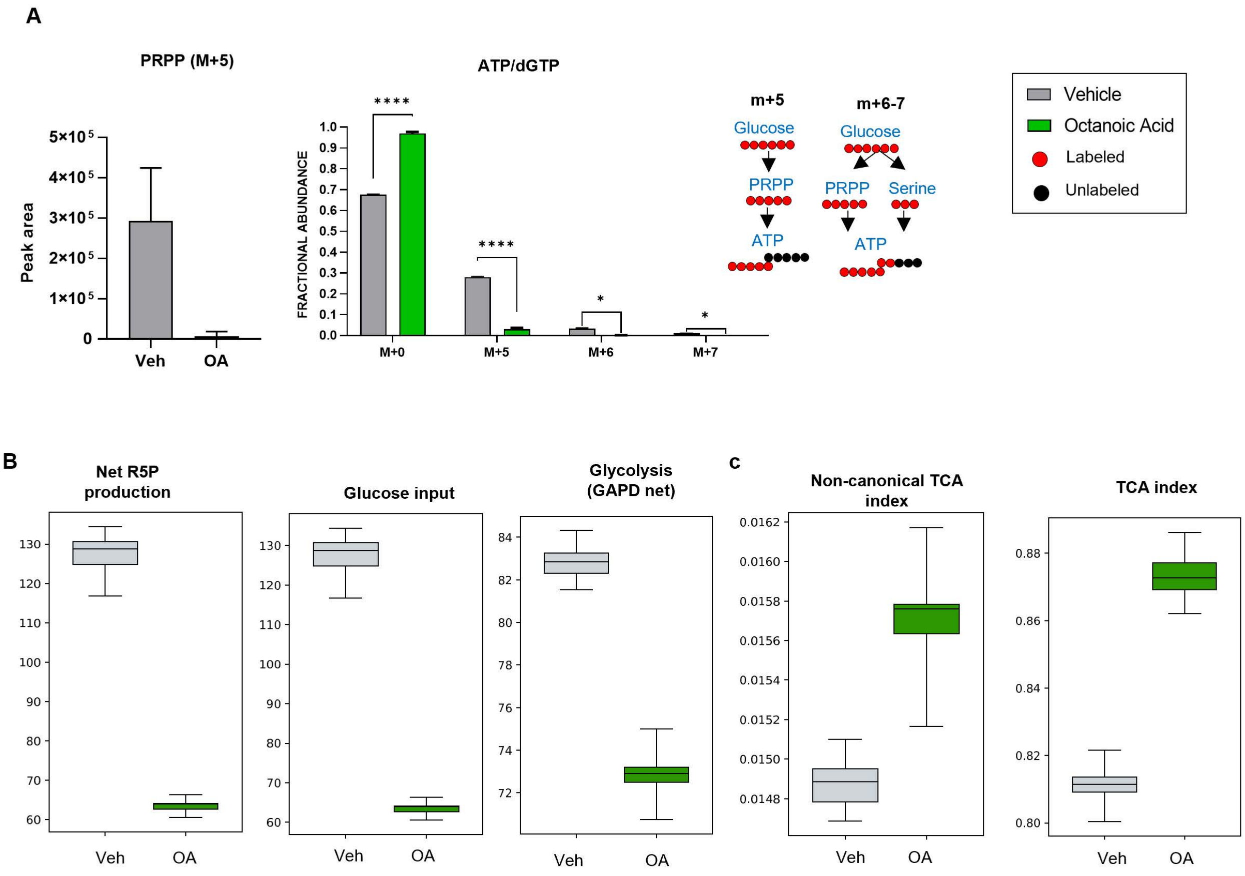

Fig. S1: ^{13}C metabolic flux analysis

A, Fractional abundance of phosphorilbosyl pyrophosphate (PRPP) M+5 and ATP isotopologues M+5-M+7. *p < 0.05, ****p < 0.0001 (2way ANOVA with Turkey test, n=3, mean with sd). 4 hours labeling. On the right schematic of derivation and contribution of carbon atoms in ATP synthesis.

B, 13C Metabolic flux calculations: Net R5P production, Glucose input, Glycolisis, Non-canonical TCA index and TCA index.

C, 13C Metabolic flux calculations: Non-canonical TCA index and TCA index.

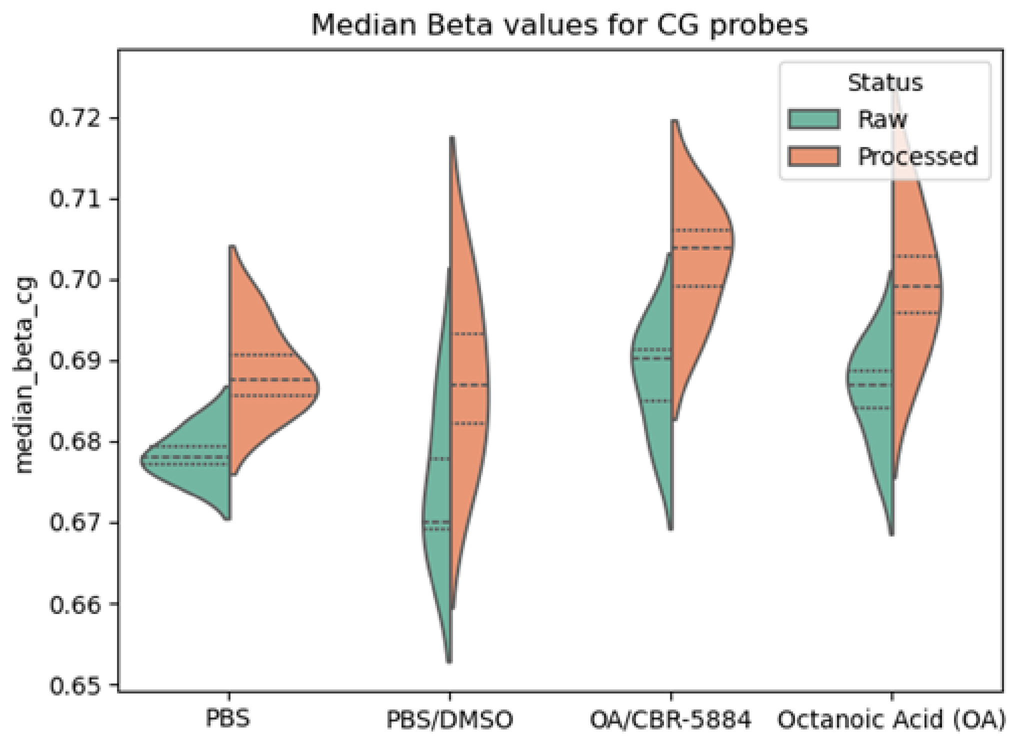

Fig. S2. DNA methylation upon Octanoic acid (OA).

Raw and processed median beta values for CpG methylation level for vehicle (PBS or PBS/DMSO), OA, or OA plus CBR-5884.

A

Gene	Gene Summary
**PCDH1**	Involved in neural cell adhesion (possible role in neuronal development).
**LORICRIN**	Major keratinocyte cell envelope protein
**MIR4652**	
**NIBAN1**	May be involved in the endoplasmic reticulum stress response.
**HEY2-AS1**	
**RAB3C**	Member of the RAS oncogene family
**TNFRSF19**	May play an essential role in embryonic development.
**LOC105371811**	
**PLAG1**	Proto-oncogene, stem cell marker
**SLC30A3**	Zinc ion transporter mediating the import of zinc from cytoplasm into synaptic vesicles
**C3orf70**	May play a role in neuronal and neurobehavioral development
**LGR6**	Genome-wide association studies identified LGR6 as an ER-negative and triple-negative-specific breast cancer germline susceptibility gene

B

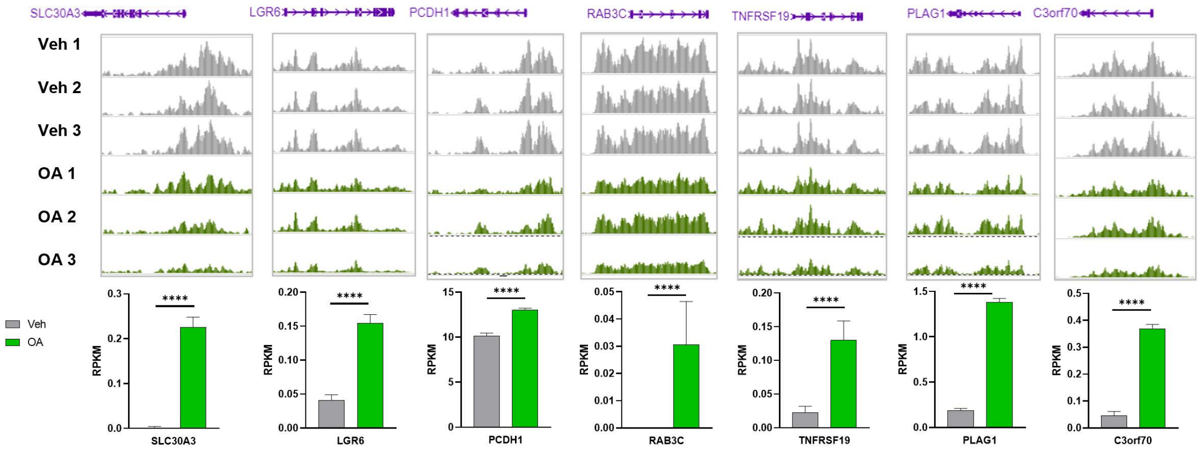

C

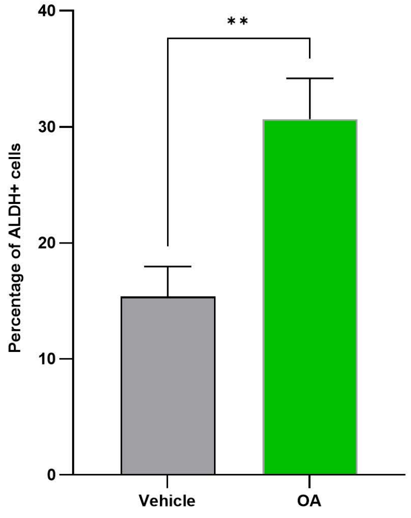

Fig. S3. CUT&RUN for H3K27me3 upon Octanoic acid (OA).

A, Table depicting all genes associated with H3K27me3 peaks. Gene summary is from UniProtKB/Swiss-Prot Summary for each gene.

B, Genes differentially modulated by OA with H3K27me3 peaks.

C, Identification of stem-like cells (ALDH+) using ALDEFLUOR test in vehicle and OA treated MCF-10A cells.

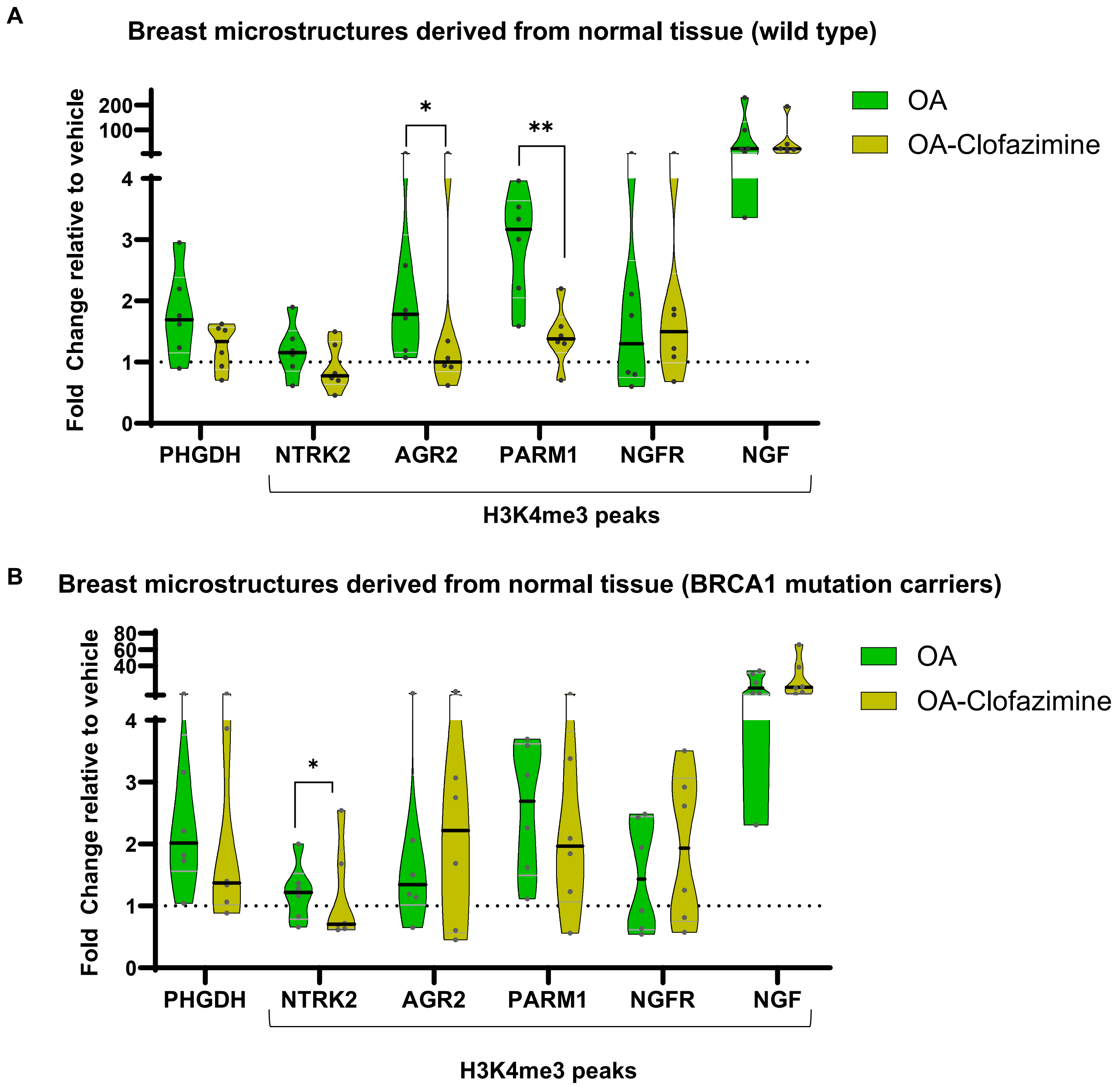

Fig. S4. Gene expression analysis of tissue derived breast microstructures.

A, Violin plots showing the expression of *PHGDH* and genes with enriched H3K4me3 peaks (*NTRK2*, *AGR2*, *PARM1*, *NGFR* and *NGF*) in tissue derived microstructures from wild type individuals. $*p < 0.05$, $**p < 0.01$ ($n = 6$, Paired t test).

B, Violin plots showing the expression of *PHGDH* and genes with enriched H3K4me3 peaks (*NTRK2*, *AGR2*, *PARM1*, *NGFR* and *NGF*) in tissue derived microstructures from BRCA1 mutation carriers. *$p < 0.05$, **$p < 0.01$ ($n = 6$, Paired t test).

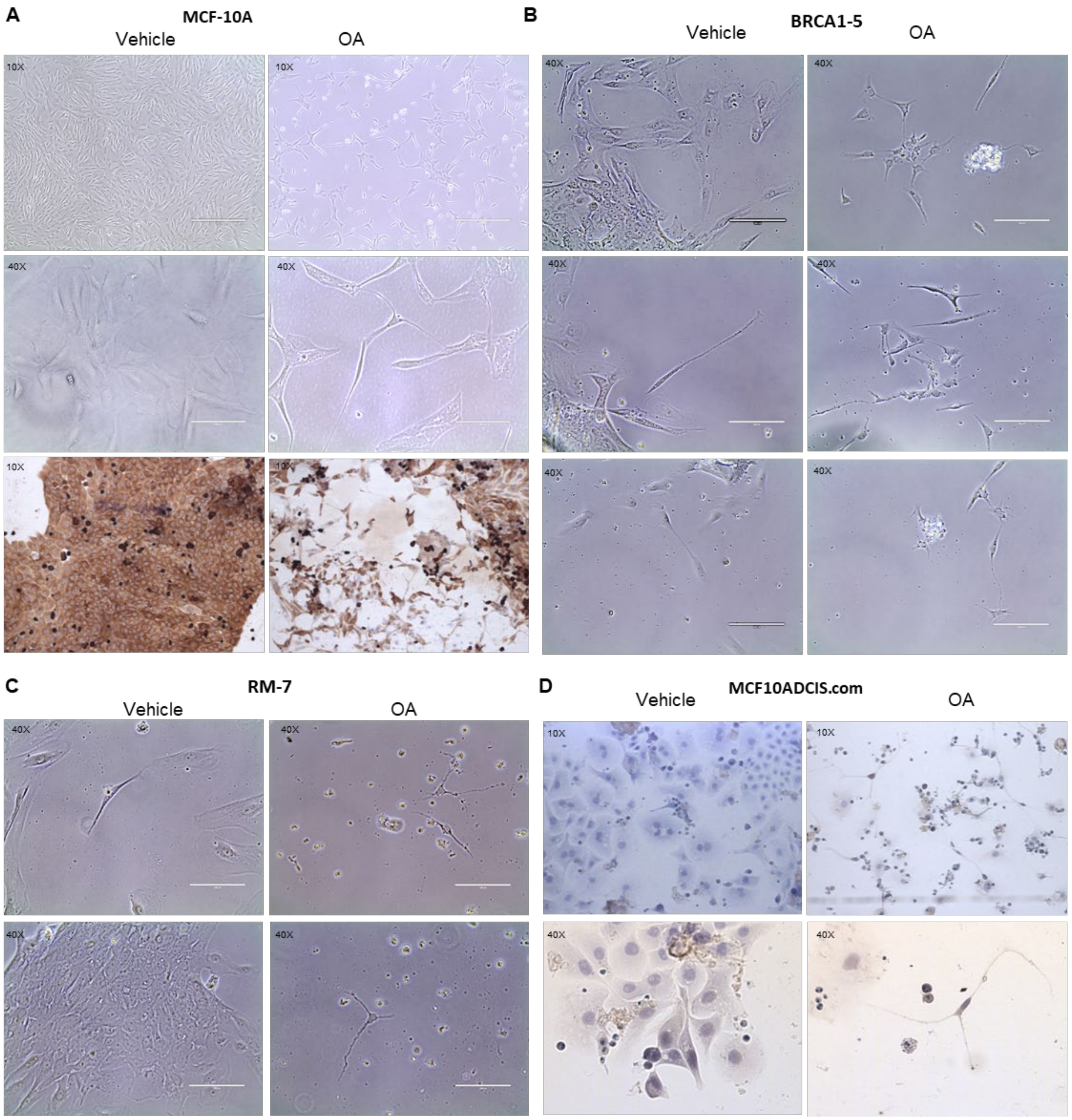

Fig. S5. Breast epithelial cells adopt a neural-like phenotype upon Octanoic acid (OA).

A, MCF-10A cells growing in PDL/L plates following exposure to vehicle (left) or 5 mM OA (right) for 1 week. Magnification: 10x (Upper), 40x (Middle). Bottom shows

Micrographs of MCF-10A cells growing in PDL/L plates following exposure to vehicle (left) or 5 mM OA (right) for 24 hrs. Magnification: 10x. PanCK and DAPI staining.

B, Primary cells from a breast microstructure derived from a BRCA1 mutation carrier growing in PDL/L plates following exposure to vehicle (left) or 5 mM OA (right) for 1 week. Magnification: 40x.

C, Primary cells from a breast microstructure derived from a wild-type donor growing in PDL/L plates following exposure to vehicle (left) or 5 mM OA (right) for 1 week. Magnification: 40x.

D, Micrographs of DCIS.com cells following exposure to vehicle (left) or 5 mM OA (right) for 24 hrs. Magnification: 10x (Upper) and 40x (Lower). DAPI staining.

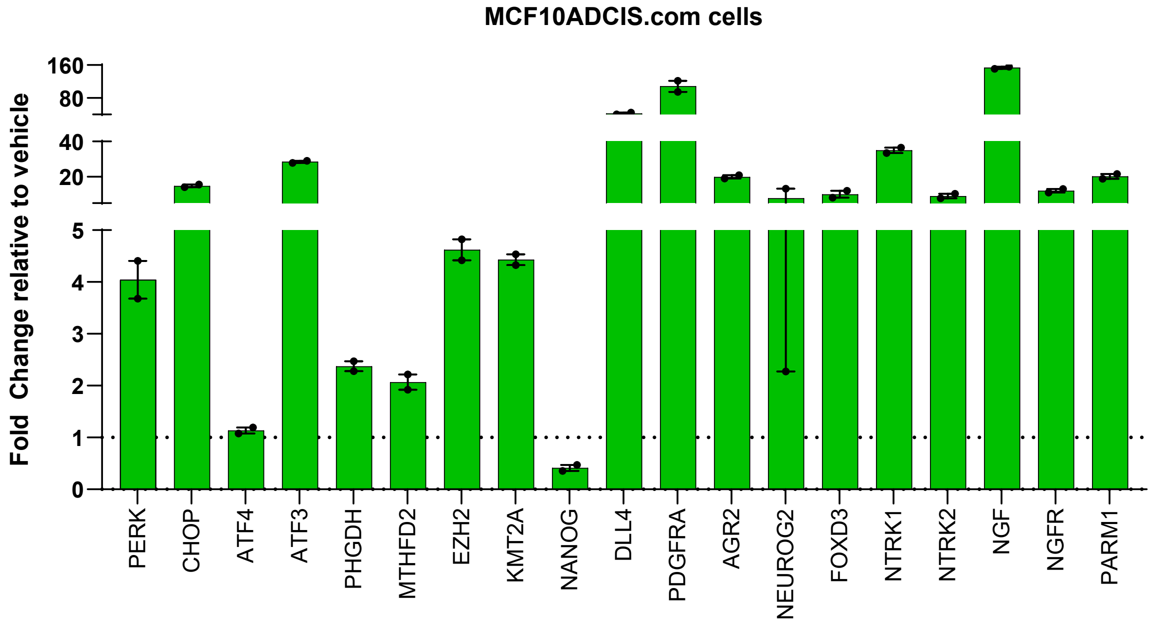

Fig. S6. Gene expression analysis of MCF10ADCIS.com cells.

Boxplots showing the expression of OA-induced genes upon OA measured by qPCR (mean ± SEM with n = 2).

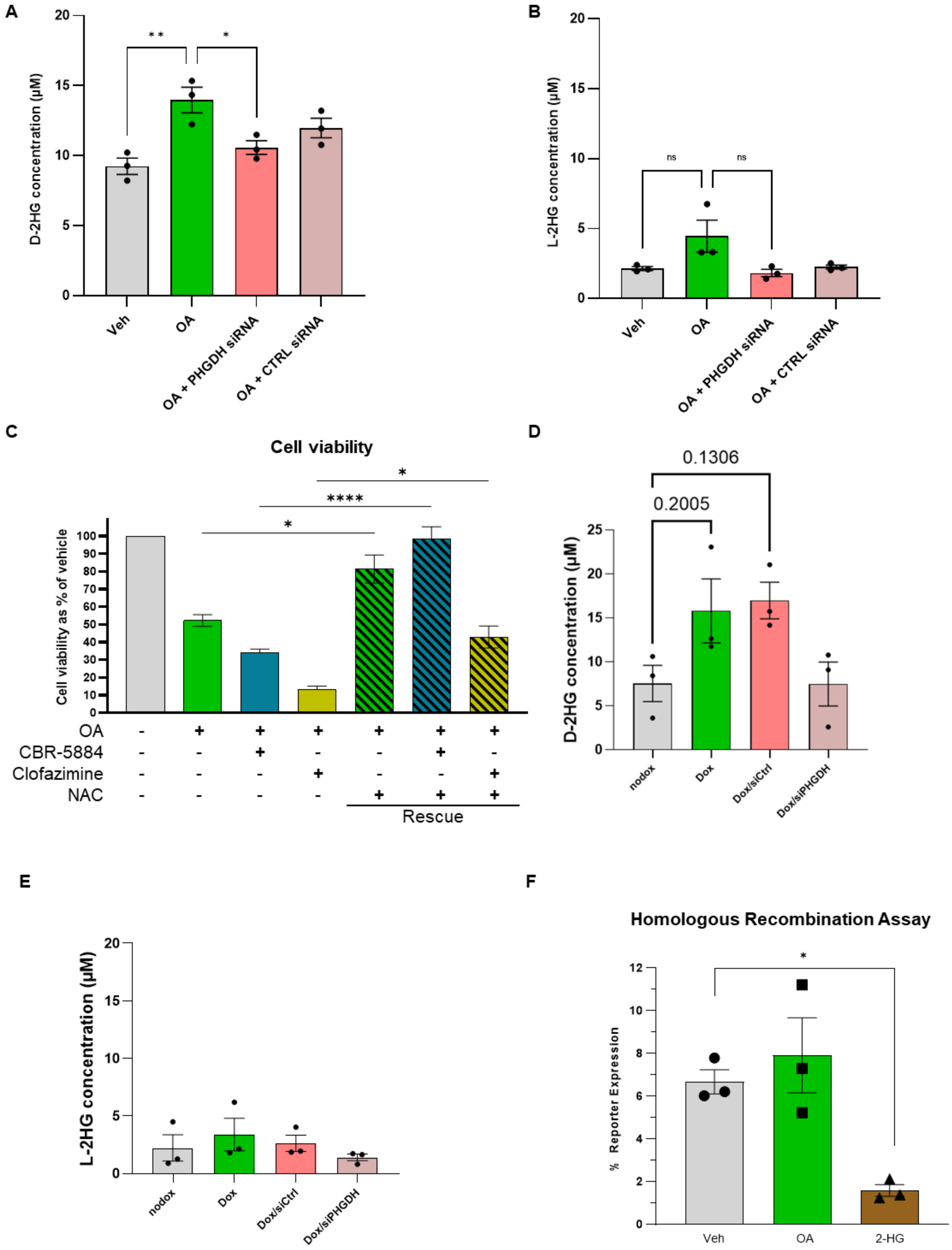
A
D-2HG concentration (µM)
20
15
10
5
0
**
*
Veh
OA
OA + PHGDH siRNA
OA + CTRL siRNA
B
L-2HG concentration (µM)
20
15
10
5
0
ns
ns
Veh
OA
OA + PHGDH siRNA
OA + CTRL siRNA
C
Cell viability
Cell viability as % of vehicle
100
90
80
70
60
50
40
30
20
10
0
*
****
*
OA - + + + + + +
CBR-5884 - - + - - + -
Clofazimine - - - + - - +
NAC - - - - + + +
Rescue
D
0.1306
0.2005
D-2HG concentration (µM)
25
20
15
10
5
0
nodox
Dox
Dox/siCtrl
Dox/siPHGDH
E
L-2HG concentration (µM)
20
15
10
5
0
nodox
Dox
Dox/siCtrl
Dox/siPHGDH
F
Homologous Recombination Assay
*
% Reporter Expression
12
10
8
6
4
2
0
Veh
OA
2-HG

Fig. S7. 2-HG, cell viability and Homologous recombination assay

A, Quantification of D-2-HG in MCF-10A cells. *$p < 0.05$, **$p < 0.01$ (2way ANOVA with Turkey test, n=3, mean with SEM).

B, Quantification of L-2-HG in MCF-10A cells. ns = not significant (2way ANOVA with Turkey test, n=3, mean with SEM).

C, Percentage of cell viability relative to the vehicle group (PBS plus DMSO) of MCF-10A cells treated with OA ± NAC, OA plus CBR-5884 ± NAC, or OA plus Clofazimine ± NAC for 24 hrs. *$p < 0.05$, ****$p < 0.0001$ (Ordinary one-way ANOVA with Turkey test, n=3, mean with SEM).

D, Quantification of D-2-HG in DOX-induced PHGDH overexpression MCF-10A cells. *$p < 0.05$, **$p < 0.01$ (2way ANOVA with Turkey test, n=3, mean with SEM).

E, Quantification of L-2-HG in DOX-induced PHGDH overexpression MCF-10A cells. ns = not significant (2way ANOVA with Turkey test, n=3, mean with SEM).

F, Repair of I-SceI nuclease induced double strand breaks. The I-SceI recognition site is located within the 5′ end of the firefly luciferase ORF and disrupts luciferase activity. A promoter-less luciferase donor DNA is located downstream as a template for HDR. The I-SceI digested luciferase reporter or control luciferase reporter were transfected into MCF-10A cells. 24 hours after transfection cells were exposed to vehicle, Octanoic acid (OA) or 900 μM 2-HG for 24 hours. Cell lysates were harvested 48 h post-transfection and assayed for luciferase activity. Bars represent mean +/− SEM. Statistical analysis by t-test (n=3).

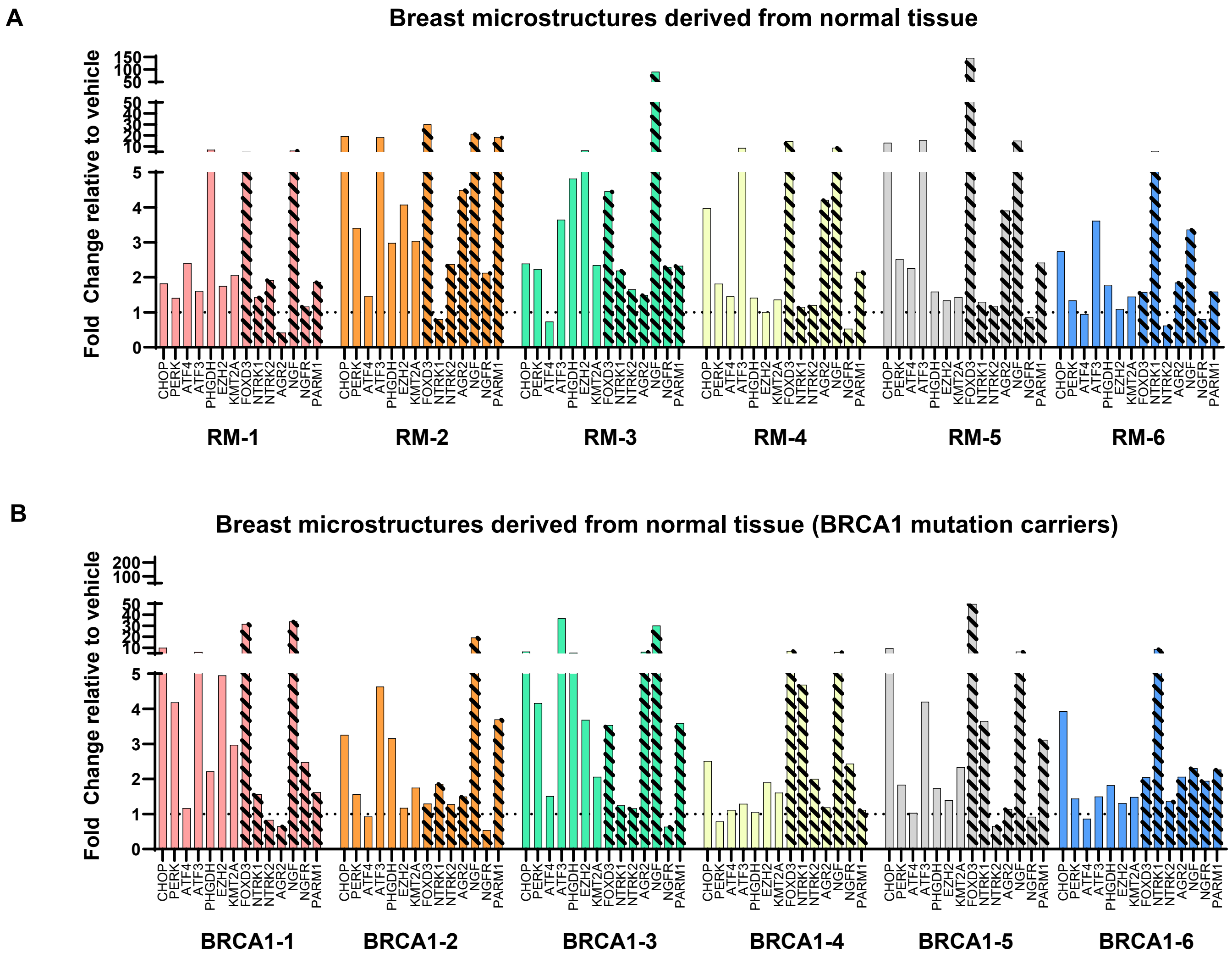

Fig. S8: Gene expression analysis of tissue derived breast microstructures.

A, Boxplots showing the expression of OA-induced genes upon OA in tissue-derived breast microstructures from 6 from wild-type donors measured by rt-qPCR. Boxplots of neural-related genes are shown with diagonal bars.

B, Boxplots showing the expression of OA-induced genes upon OA in tissue-derived breast microstructures from 6 BRCA1 mutation carriers measured by rt-qPCR. Boxplots of neural-related genes are shown with diagonal bars.

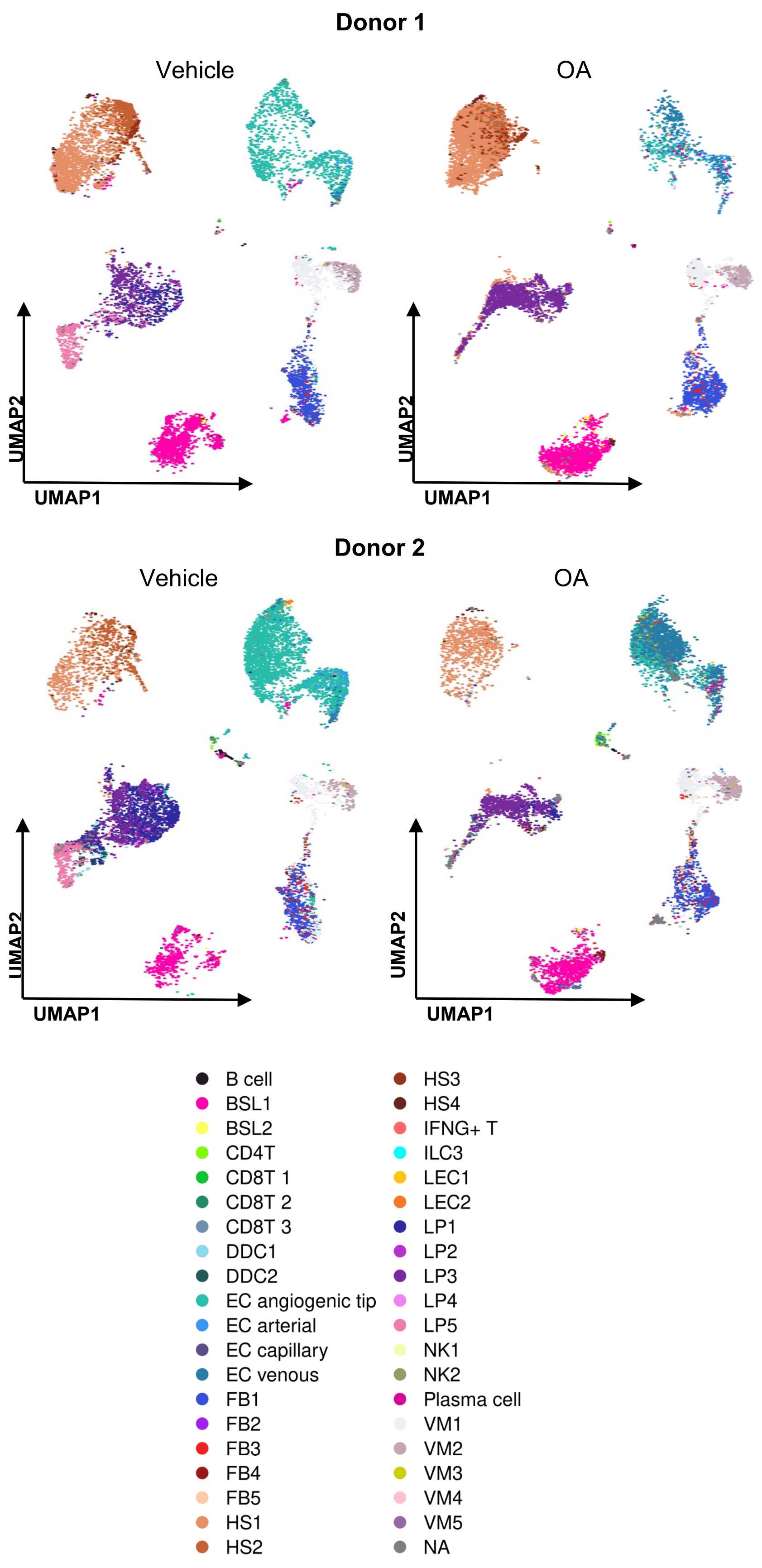

	Donor 1		Donor 2	
	Vehicle	OA	Vehicle	OA
**BSL1***	1425	1640	620	995
**BSL2**	13	25	3	9
**HS1***	1742	4799	629	852
**HS2**	491	201	364	9
**HS3**	90	134	8	0
**HS4**	35	42	28	52
**LP1**	390	67	1713	203
**LP2**	45	44	44	41
**LP3***	427	1231	594	729
**LP4**	1	0	18	0
**LP5**	414	0	409	1
**FB1**	680	822	775	798
**FB2**	2	0	8	2
**FB3**	12	46	67	80
**FB4**	7	0	31	33
**FB5**	11	27	18	80
**VM1**	458	492	359	515
**VM2**	180	225	124	279
**VM3**	1	0	2	1
**VM4**	1	0	1	0
**VM5**	1	1	1	0
**EC angiogenic tip**	1137	211	3007	1675
**EC arterial**	27	132	73	145
**EC capillary**	1	0	3	1
**EC venous**	49	314	75	1519
**B cell**	2	5	52	15
**CD4T**	2	2	21	159
**CD8T 1**	1	0	11	39
**CD8T 2**	0	0	2	26
**CD8T 3**	1	0	5	4
**IFNG+ T**	0	0	0	1
**ILC3**	0	0	0	2
**LEC1**	1	0	22	15
**LEC2**	1	0	9	0
**NK1**	0	0	1	2
**NK2**	0	0	0	1
**Plasma cell**	1	17	19	37

Fig. S9: Approximation and Projection (UMAP) plot.

UMAP plot shows the approximation and projection of cells from two donors. Cell subtypes are defined by Reed et al. (*65*). The table displays the number of cells per subtype: green indicates subtypes that increased with OA, orange indicates subtypes that decreased with OA, and grey indicates subtypes that showed no change. *Epithelial subtypes selected for metabolic flux pathway analyses.

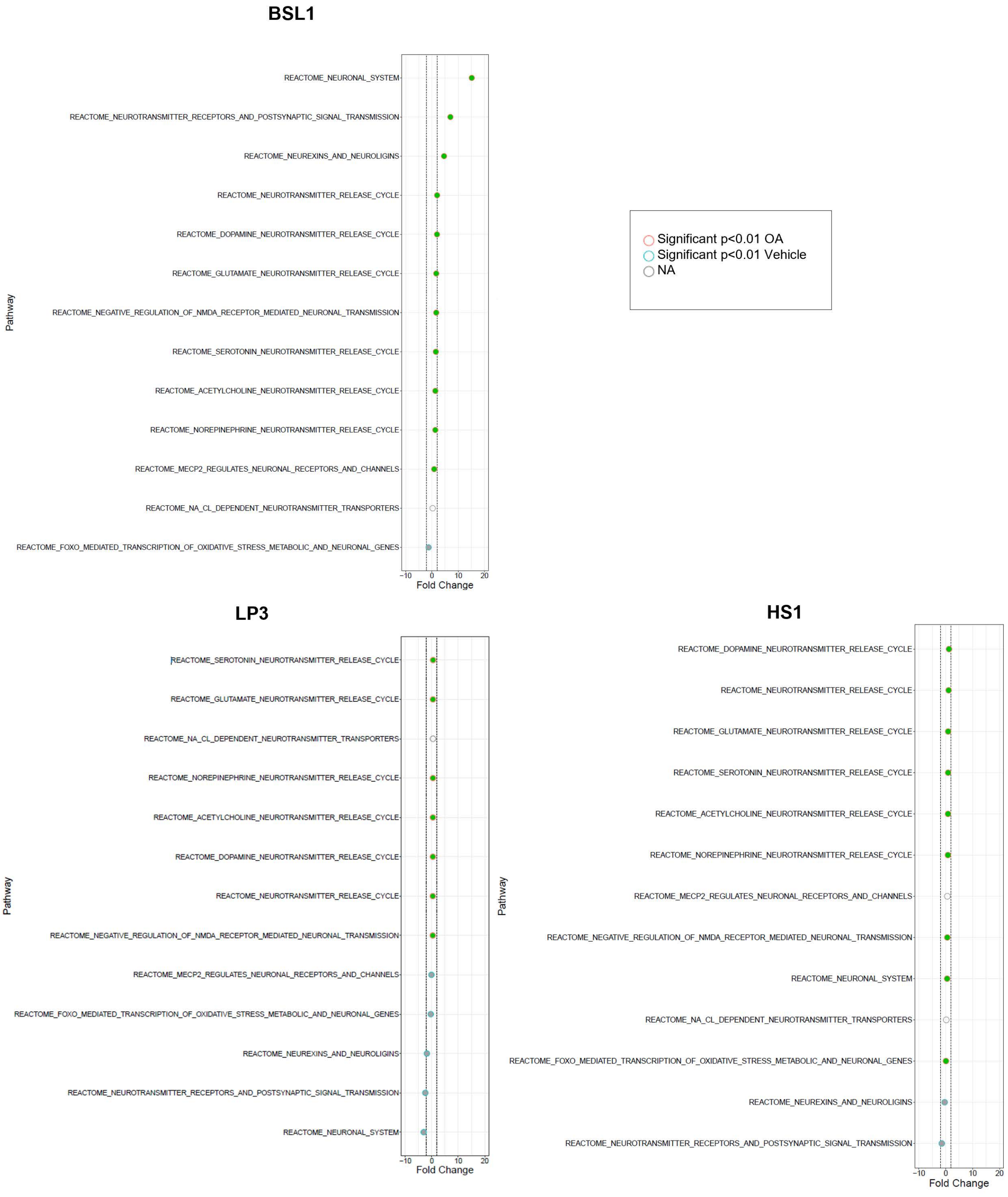

Fig. S10: Single cell pathway analysis.

Single cell pathway analysis plots showing neural-related terms in BSL1, HS1 and LP3.

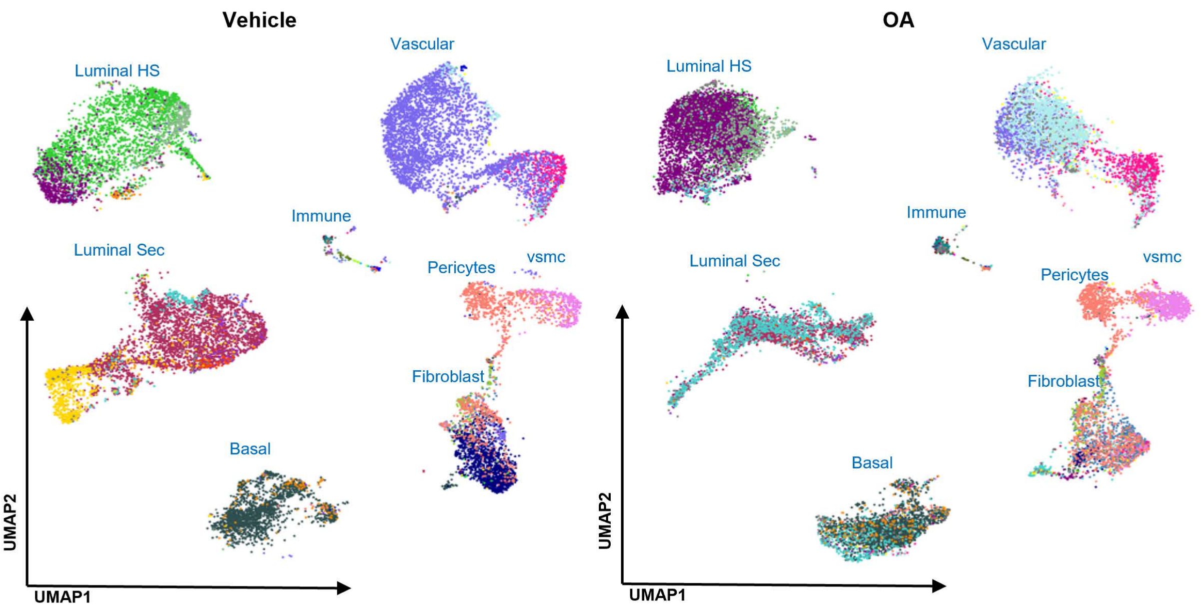

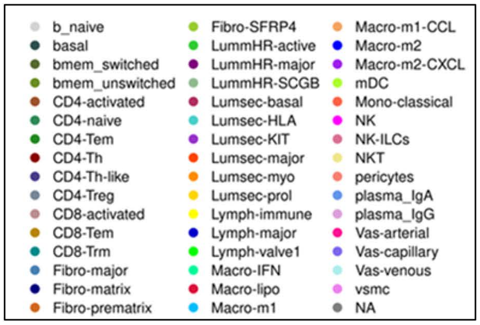

Cell subtype	% (vehicle)	% (OA)	Cell subtype	% (vehicle)	% (OA)	Cell subtype	% (vehicle)	% (OA)
basal	10.5	11.2	b_naive	0.01	0.00	Macro-lipo	0.07	0.01
LummHR-active	13.5	6.1	bmem_switched	0.06	0.04	Macro-m1	0.05	0.02
LummHR-major	3.5	20.3	bmem_unswitched	0.06	0.01	Macro-m1-CCL	0.02	0.08
LummHR-SCGB	2.1	3.2	CD4-activated	0.03	0.00	Macro-m2	0.11	0.04
Lumsec-basal	19.4	8.5	CD4-naive	0.03	0.40	Macro-m2-CXCL	0.00	0.02
Lumsec-HLA	1.1	9.4	CD4-Tem	0.00	0.01	mDC	0.04	0.00
Lumsec-KIT	0.5	0.2	CD4-Th	0.06	0.23	Mono-classical	0.03	0.06
Lumsec-major	0.5	0.1	CD4-Th-like	0.04	0.21	NK	0.00	0.01
Lumsec-myo	1.0	0.8	CD4-Treg	0.02	0.04	NK-ILCs	0.00	0.01
Lumsec-prol	5.1	0.0	CD8-activated	0.01	0.01	NKT	0.00	0.01
Fibro-major	0.7	4.5	CD8-Tem	0.00	0.01	plasma_IgA	0.08	0.18
Fibro-matrix	7.6	0.4	CD8-Trm	0.06	0.30	plasma_IgG	0.01	0.03
Fibro-prematrix	0.0	0.0	Lymph-immune	0.14	1.21	Vas-arterial	3.32	5.54
Fibro-SFRP4	0.7	0.9	Lymph-major	0.14	0.20	Vas-capillary	20.90	4.65
pericytes	6.2	8.2	Lymph-valve1	0.01	0.01	Vas-venous	0.89	9.63
			Macro-IFN	0.01	0.01	vsmc	1.60	3.24

Fig. S11: Approximation and Projection (UMAP) plot.

Approximation and Projection (UMAP) plot of 36’904 cells identified a total of 25 different cell clusters. Cell subtypes defined by Kumar *et al*. (*23*). Table shows cell subtypes proportions.

Legends for Supplementary Tables

Table S1: Differentially enriched CUT&RUN peaks**.**

A. Differentially enriched CUT&RUN peaks for H3K27me3 between vehicle and OA treatment.

B. Differentially enriched CUT&RUN peaks for H3K4me3 between vehicle and OA treatment.

Table S2: Differential expression analysis using single-cell data.

Table S3: Single cell pathway analysis.

Table S4: List of the qPCR probe assays and raw CT values for Fig. 2F-G and Fig. S6.

Table S5: List of the qPCR probe assays and raw CT values for Fig. S4A-B and Fig. S8A-B.

Table S6: Raw peak area values from isotope tracing in MCF-10A cells.

Table S7: Raw peak area values from targeted metabolomics analysis of MCF-10A cells.

Table S8: Compass prediction of differentially active metabolic reactions between OA and vehicle treatment in BSL1.

Table S9: Compass prediction of differentially active metabolic reactions between OA and vehicle treatment in LP3.

Table S10: Compass prediction of differentially active metabolic reactions between OA and vehicle treatment in HS1.

REFERENCES AND NOTES

1. S. M. Lima, R. D. Kehm, M. B. Terry, Global breast cancer incidence and mortality trends by region, age-groups, and fertility patterns. *EClinicalMedicine* **38**, 100985 (2021).

2. Global Burden of Disease 2019 Cancer Collaboration, Cancer incidence, mortality, years of life lost, years lived with disability, and disability-adjusted life years for 29 cancer groups from 2010 to 2019: A systematic analysis for the global burden of disease study 2019. *JAMA Oncol.* **8**, 420–444 (2022).

3. M. B. Sporn, Approaches to prevention of epithelial cancer during the preneoplastic period. *Cancer Res.* **36**, 2699–2702 (1976).

4. X. R. Yang, J. Chang-Claude, E. L. Goode, F. J. Couch, H. Nevanlinna, R. L. Milne, M. Gaudet, M. K. Schmidt, A. Broeks, A. Cox, P. A. Fasching, R. Hein, A. B. Spurdle, F. Blows, K. Driver, D. Flesch-Janys, J. Heinz, P. Sinn, A. Vrieling, T. Heikkinen, K. Aittomäki, P. Heikkilä, C. Blomqvist, J. Lissowska, B. Peplonska, S. Chanock, J. Figueroa, L. Brinton, P. Hall, K. Czene, K. Humphreys, H. Darabi, J. Liu, L. J. van 't Veer, F. E. van Leeuwen, I. L. Andrulis, G. Glendon, J. A. Knight, A. M. Mulligan, F. P. O'Malley, N. Weerasooriya, E. M. John, M. W. Beckmann, A. Hartmann, S. B. Weihbrecht, D. L. Wachter, S. M. Jud, C. R. Loehberg, L. Baglietto, D. R. English, G. G. Giles, C. A. McLean, G. Severi, D. Lambrechts, T. Vandorpe, C. Weltens, R. Paridaens, A. Smeets, P. Neven, H. Wildiers, X. Wang, J. E. Olson, V. Cafourek, Z. Fredericksen, M. Kosel, C. Vachon, H. E. Cramp, D. Connley, S. S. Cross, S. P. Balasubramanian, M. W. Reed, T. Dörk, M. Bremer, A. Meyer, J. H. Karstens, A. Ay, T. W. Park-Simon, P. Hillemanns, J. I. Arias Pérez, P. Menéndez Rodríguez, P. Zamora, J. Benítez, Y. D. Ko, H. P. Fischer, U. Hamann, B. Pesch, T. Brüning, C. Justenhoven, H. Brauch, D. M. Eccles, W. J. Tapper, S. M. Gerty, E. J. Sawyer, I. P. Tomlinson, A. Jones, M. Kerin, N. Miller, N. McInerney, H. Anton-Culver, A. Ziogas, C. Y. Shen, C. N. Hsiung, P. E. Wu, S. L. Yang, J. C. Yu, S. T. Chen, G. C. Hsu, C. A. Haiman, B. E. Henderson, L. Le Marchand, L. N. Kolonel, A. Lindblom, S. Margolin, A. Jakubowska, J. Lubiński, T. Huzarski, T. Byrski, B. Górski, J. Gronwald, M. J. Hooning, A. Hollestelle, A. M. van den Ouweland, A. Jager, M. Kriege, M. M. Tilanus-Linthorst, M. Collée, S. Wang-Gohrke, K. Pylkäs, A. Jukkola-Vuorinen, K. Mononen, M. Grip, P. Hirvikoski, R. Winqvist, A. Mannermaa, V. M. Kosma, J. Kauppinen, V. Kataja, P. Auvinen, Y. Soini, R. Sironen, S. E. Bojesen, D. D. Ørsted, D. Kaur-

Knudsen, H. Flyger, B. G. Nordestgaard, H. Holland, G. Chenevix-Trench, S. Manoukian, M. Barile, P. Radice, S. E. Hankinson, D. J. Hunter, R. Tamimi, S. Sangrajrang, P. Brennan, J. McKay, F. Odefrey, V. Gaborieau, P. Devilee, P. E. Huijts, R. A. Tollenaar, C. Seynaeve, G. S. Dite, C. Apicella, J. L. Hopper, F. Hammet, H. Tsimiklis, L. D. Smith, M. C. Southey, M. K. Humphreys, D. Easton, P. Pharoah, M. E. Sherman, M. Garcia-Closas, Associations of breast cancer risk factors with tumor subtypes: A pooled analysis from the Breast Cancer Association Consortium studies.*J. Natl. Cancer Inst.* **103**, 250–263 (2011).

5. R. L. Milne, K. B. Kuchenbaecker, K. Michailidou, J. Beesley, S. Kar, S. Lindström, S. Hui, A. Lemaçon, P. Soucy, J. Dennis, X. Jiang, A. Rostamianfar, H. Finucane, M. K. Bolla, L. McGuffog, Q. Wang, C. M. Aalfs, M. Adams, J. Adlard, S. Agata, S. Ahmed, H. Ahsan, K. Aittomäki, F. Al-Ejeh, J. Allen, C. B. Ambrosone, C. I. Amos, I. L. Andrulis, H. Anton-Culver, N. N. Antonenkova, V. Arndt, N. Arnold, K. J. Aronson, B. Auber, P. L. Auer, M. Ausems, J. Azzollini, F. Bacot, J. Balmaña, M. Barile, L. Barjhoux, R. B. Barkardottir, M. Barrdahl, D. Barnes, D. Barrowdale, C. Baynes, M. W. Beckmann, J. Benitez, M. Bermisheva, L. Bernstein, Y. J. Bignon, K. R. Blazer, M. J. Blok, C. Blomqvist, W. Blot, K. Bobolis, B. Boeckx, N. V. Bogdanova, A. Bojesen, S. E. Bojesen, B. Bonanni, A. L. Børresen-Dale, A. Bozsik, A. R. Bradbury, J. S. Brand, H. Brauch, H. Brenner, B. B.-d. Paillerets, C. Brewer, L. Brinton, P. Broberg, A. Brooks-Wilson, J. Brunet, T. Brüning, B. Burwinkel, S. S. Buys, J. Byun, Q. Cai, T. Caldés, M. A. Caligo, I. Campbell, F. Canzian, O. Caron, A. Carracedo, B. D. Carter, J. E. Castelao, L. Castera, V. Caux-Moncoutier, S. B. Chan, J. Chang-Claude, S. J. Chanock, X. Chen, T. D. Cheng, J. Chiquette, H. Christiansen, K. B. M. Claes, C. L. Clarke, T. Conner, D. M. Conroy, J. Cook, E. Cordina-Duverger, S. Cornelissen, I. Coupier, A. Cox, D. G. Cox, S. S. Cross, K. Cuk, J. M. Cunningham, K. Czene, M. B. Daly, F. Damiola, H. Darabi, R. Davidson, K. De Leeneer, P. Devilee, E. Dicks, O. Diez, Y. C. Ding, N. Ditsch, K. F. Doheny, S. M. Domchek, C. M. Dorfling, T. Dörk, I. Dos-Santos-Silva, S. Dubois, P. A. Dugué, M. Dumont, A. M. Dunning, L. Durcan, M. Dwek, B. Dworniczak, D. Eccles, R. Eeles, H. Ehrencrona, U. Eilber, B. Ejlertsen, A. B. Ekici, A. H. Eliassen, C. Engel, M. Eriksson, L. Fachal, L. Faivre, P. A. Fasching, U. Faust, J. Figueroa, D. Flesch-Janys, O. Fletcher, H. Flyger, W. D. Foulkes, E. Friedman, L. Fritschi, D. Frost, M. Gabrielson, P. Gaddam, M. D. Gammon, P. A. Ganz, S. M. Gapstur, J. Garber, V. Garcia-Barberan, J. A. García-Sáenz, M. M. Gaudet, M. Gauthier-Villars, A. Gehrig, V. Georgoulias, A. M. Gerdes, G. G. Giles, G. Glendon, A. K. Godwin, M. S. Goldberg, D. E. Goldgar, A. González-Neira, P.

Goodfellow, M. H. Greene, G. I. G. Alnæs, M. Grip, J. Gronwald, A. Grundy, D. Gschwantler-Kaulich, P. Guénel, Q. Guo, L. Haeberle, E. Hahnen, C. A. Haiman, N. Håkansson, E. Hallberg, U. Hamann, N. Hamel, S. Hankinson, T. V. O. Hansen, P. Harrington, S. N. Hart, J. M. Hartikainen, C. S. Healey, A. Hein, S. Helbig, A. Henderson, J. Heyworth, B. Hicks, P. Hillemanns, S. Hodgson, F. B. Hogervorst, A. Hollestelle, M. J. Hooning, B. Hoover, J. L. Hopper, C. Hu, G. Huang, P. J. Hulick, K. Humphreys, D. J. Hunter, E. N. Imyanitov, C. Isaacs, M. Iwasaki, L. Izatt, A. Jakubowska, P. James, R. Janavicius, W. Janni, U. B. Jensen, E. M. John, N. Johnson, K. Jones, M. Jones, A. Jukkola-Vuorinen, R. Kaaks, M. Kabisch, K. Kaczmarek, D. Kang, K. Kast, R. Keeman, M. J. Kerin, C. M. Kets, M. Keupers, S. Khan, E. Khusnutdinova, J. I. Kiiski, S. W. Kim, J. A. Knight, I. Konstantopoulou, V. M. Kosma, V. N. Kristensen, T. A. Kruse, A. Kwong, A. V. Lænkholm, Y. Laitman, F. Lalloo, D. Lambrechts, K. Landsman, C. Lasset, C. Lazaro, L. Le Marchand, J. Lecarpentier, A. Lee, E. Lee, J. W. Lee, M. H. Lee, F. Lejbkowicz, F. Lesueur, J. Li, J. Lilyquist, A. Lincoln, A. Lindblom, J. Lissowska, W. Y. Lo, S. Loibl, J. Long, J. T. Loud, J. Lubinski, C. Luccarini, M. Lush, R. J. MacInnis, T. Maishman, E. Makalic, I. M. Kostovska, K. E. Malone, S. Manoukian, J. E. Manson, S. Margolin, J. W. M. Martens, M. E. Martinez, K. Matsuo, D. Mavroudis, S. Mazoyer, C. McLean, H. Meijers-Heijboer, P. Menéndez, J. Meyer, H. Miao, A. Miller, N. Miller, G. Mitchell, M. Montagna, K. Muir, A. M. Mulligan, C. Mulot, S. Nadesan, K. L. Nathanson, S. L. Neuhausen, H. Nevanlinna, I. Nevelsteen, D. Niederacher, S. F. Nielsen, B. G. Nordestgaard, A. Norman, R. L. Nussbaum, E. Olah, O. I. Olopade, J. E. Olson, C. Olswold, K. R. Ong, J. C. Oosterwijk, N. Orr, A. Osorio, V. S. Pankratz, L. Papi, T. W. Park-Simon, Y. Paulsson-Karlsson, R. Lloyd, I. S. Pedersen, B. Peissel, A. Peixoto, J. I. A. Perez, P. Peterlongo, J. Peto, G. Pfeiler, C. M. Phelan, M. Pinchev, D. Plaseska-Karanfilska, B. Poppe, M. E. Porteous, R. Prentice, N. Presneau, D. Prokofieva, E. Pugh, M. A. Pujana, K. Pylkäs, B. Rack, P. Radice, N. Rahman, J. Rantala, C. Rappaport-Fuerhauser, G. Rennert, H. S. Rennert, V. Rhenius, K. Rhiem, A. Richardson, G. C. Rodriguez, A. Romero, J. Romm, M. A. Rookus, A. Rudolph, T. Ruediger, E. Saloustros, J. Sanders, D. P. Sandler, S. Sangrajrang, E. J. Sawyer, D. F. Schmidt, M. J. Schoemaker, F. Schumacher, P. Schürmann, L. Schwentner, C. Scott, R. J. Scott, S. Seal, L. Senter, C. Seynaeve, M. Shah, P. Sharma, C. Y. Shen, X. Sheng, H. Shimelis, M. J. Shrubsole, X. O. Shu, L. E. Side, C. F. Singer, C. Sohn, M. C. Southey, J. J. Spinelli, A. B. Spurdle, C. Stegmaier, D. Stoppa-Lyonnet, G. Sukiennicki, H. Surowy, C. Sutter, A. Swerdlow, C. I. Szabo, R. M. Tamimi, Y. Y. Tan, J. A. Taylor, M. I. Tejada, M. Tengström, S.

H. Teo, M. B. Terry, D. C. Tessier, A. Teulé, K. Thöne, D. L. Thull, M. G. Tibiletti, L. Tihomirova, M. Tischkowitz, A. E. Toland, R. Tollenaar, I. Tomlinson, L. Tong, D. Torres, M. Tranchant, T. Truong, K. Tucker, N. Tung, J. Tyrer, H. U. Ulmer, C. Vachon, C. J. van Asperen, D. Van Den Berg, A. M. W. van den Ouweland, E. J. van Rensburg, L. Varesco, R. Varon-Mateeva, A. Vega, A. Viel, J. Vijai, D. Vincent, J. Vollenweider, L. Walker, Z. Wang, S. Wang-Gohrke, B. Wappenschmidt, C. R. Weinberg, J. N. Weitzel, C. Wendt, J. Wesseling, A. S. Whittemore, J. T. Wijnen, W. Willett, R. Winqvist, A. Wolk, A. H. Wu, L. Xia, X. R. Yang, D. Yannoukakos, D. Zaffaroni, W. Zheng, B. Zhu, A. Ziogas, E. Ziv, K. K. Zorn, M. Gago-Dominguez, A. Mannermaa, H. Olsson, M. R. Teixeira, J. Stone, K. Offit, L. Ottini, S. K. Park, M. Thomassen, P. Hall, A. Meindl, R. K. Schmutzler, A. Droit, G. D. Bader, P. D. P. Pharoah, F. J. Couch, D. F. Easton, P. Kraft, G. Chenevix-Trench, M. García-Closas, M. K. Schmidt, A. C. Antoniou, J. Simard, Identification of ten variants associated with risk of estrogen-receptor-negative breast cancer. *Nat. Genet.* **49**, 1767–1778 (2017).

6. N. C. Turner, J. S. Reis-Filho, A. M. Russell, R. J. Springall, K. Ryder, D. Steele, K. Savage, C. E. Gillett, F. C. Schmitt, A. Ashworth, A. N. Tutt, BRCA1 dysfunction in sporadic basal-like breast cancer. *Oncogene* **26**, 2126–2132 (2007).

7. K. C. Chu, W. F. Anderson, A. Fritz, L. A. Ries, O. W. Brawley, Frequency distributions of breast cancer characteristics classified by estrogen receptor and progesterone receptor status for eight racial/ethnic groups. *Cancer* **92**, 37–45 (2001).

8. M. V. A. dos Santos, I. Silva, Breast density and parenchymal patterns as markers of breast cancer risk: A meta-analysis. *Cancer Epidemiol. Biomarkers Prev.* **15**, 1159–1169 (2006).

9. W. D. Dupont, D. L. Page, Risk factors for breast cancer in women with proliferative breast disease. *N. Engl. J. Med.* **312**, 146–151 (1985).

10. C. Bouchardy, S. Benhamou, G. Fioretta, H. M. Verkooijen, P. O. Chappuis, I. Neyroud-Caspar, M. Castiglione, V. Vinh-Hung, G. Vlastos, E. Rapiti, Risk of second breast cancer according to estrogen receptor status and family history. *Breast Cancer Res. Treat.* **127**, 233–241 (2011).

11. S. M. Swain, J. W. Wilson, E. P. Mamounas, J. Bryant, D. L. Wickerham, B. Fisher, S. Paik, N. Wolmark, Estrogen receptor status of primary breast cancer is predictive of estrogen receptor status of contralateral breast cancer. *J. Natl. Cancer Inst.* **96**, 516–523 (2004).

12. J. Wang, D. Scholtens, M. Holko, D. Ivancic, O. Lee, H. Hu, R. T. Chatterton Jr., M. E. Sullivan, N. Hansen, K. Bethke, C. M. Zalles, S. A. Khan, Lipid metabolism genes in contralateral unaffected breast and estrogen receptor status of breast cancer. *Cancer Prev. Res.* **6**, 321–330 (2013).

13. J. Wang, A. Shidfar, D. Ivancic, M. Ranjan, L. Liu, M. R. Choi, V. Parimi, D. B. Gursel, M. E. Sullivan, M. S. Najor, A. M. Abukhdeir, D. Scholtens, S. A. Khan, Overexpression of lipid metabolism genes and PBX1 in the contralateral breasts of women with estrogen receptor-negative breast cancer. *Int. J. Cancer* **140**, 2484–2497 (2017).

14. S. Yadav, R. Virk, C. H. Chung, M. Bustamante Eduardo, D. VanDerway, D. Chen, K. Burdett, H. Gao, Z. Zeng, M. Ranjan, G. Cottone, X. Xuei, S. Chandrasekaran, V. Backman, R. Chatterton, S. A. Khan, S. E. Clare, Lipid exposure activates gene expression changes associated with estrogen receptor negative breast cancer. *NPJ Breast Cancer* **8**, 59 (2022).

15. J. Fan, X. Teng, L. Liu, K. R. Mattaini, R. E. Looper, M. G. Vander Heiden, J. D. Rabinowitz, Human phosphoglycerate dehydrogenase produces the oncometabolite D-2-hydroxyglutarate. *ACS Chem. Biol.* **10**, 510–516 (2015).

16. W. Xu, H. Yang, Y. Liu, Y. Yang, P. Wang, S. H. Kim, S. Ito, C. Yang, P. Wang, M. T. Xiao, L. X. Liu, W. Q. Jiang, J. Liu, J. Y. Zhang, B. Wang, S. Frye, Y. Zhang, Y. H. Xu, Q. Y. Lei, K. L. Guan, S. M. Zhao, Y. Xiong, Oncometabolite 2-hydroxyglutarate is a competitive inhibitor of α-ketoglutarate-dependent dioxygenases. *Cancer Cell* **19**, 17–30 (2011).

17. E. Y. Chen, C. M. Tan, Y. Kou, Q. Duan, Z. Wang, G. V. Meirelles, N. R. Clark, A. Ma'ayan, Enrichr: Interactive and collaborative HTML5 gene list enrichment analysis tool. *BMC Bioinformatics* **14**, 128 (2013).

18. W. Sun, R. Liu, X. Gao, Z. Lin, H. Tang, H. Cui, E. Zhao, Targeting serine-glycine-one-carbon metabolism as a vulnerability in cancers. *Biomark Res.* **11**, 48 (2023).

19. E. McDonnell, S. B. Crown, D. B. Fox, B. Kitir, O. R. Ilkayeva, C. A. Olsen, P. A. Grimsrud, M. D. Hirschey, Lipids reprogram metabolism to become a major carbon source for histone acetylation. *Cell Rep.* **17**, 1463–1472 (2016).

20. J. C. A. de la Pérez, J. M. L, astra, F. J. Plou, E. Pérez-Lebeña, The chemistry of reactive oxygen species (ROS) revisited: Outlining their role in biological macromolecules (DNA, lipids and proteins) and induced pathologies. *Int. J. Mol. Sci.* **22**, 4642 (2021).

21. P. L. Sulkowski, C. D. Corso, N. D. Robinson, S. E. Scanlon, K. R. Purshouse, H. Bai, Y. Liu, R. K. Sundaram, D. C. Hegan, N. R. Fons, G. A. Breuer, Y. Song, K. Mishra-Gorur, H. M. De Feyter, R. A. de Graaf, Y. V. Surovtseva, M. Kachman, S. Halene, M. Günel, P. M. Glazer, R. S. Bindra, 2-Hydroxyglutarate produced by neomorphic IDH mutations suppresses homologous recombination and induces PARP inhibitor sensitivity. *Sci. Transl. Med.* **9**, eaal2463 (2017).

22. A. L. Cartaxo, M. F. Estrada, G. Domenici, R. Roque, F. Silva, E. J. Gualda, P. Loza-Alvarez, G. Sflomos, C. Brisken, P. M. Alves, S. André, C. Brito, A novel culture method that sustains ERα signaling in human breast cancer tissue microstructures. *J. Exp. Clin. Cancer Res.* **39**, 161 (2020).

23. T. Kumar, K. Nee, R. Wei, S. He, Q. H. Nguyen, S. Bai, K. Blake, M. Pein, Y. Gong, E. Sei, M. Hu, A. K. Casasent, A. Thennavan, J. Li, T. Tran, K. Chen, B. Nilges, N. Kashikar, O. Braubach, B. Ben Cheikh, N. Nikulina, H. Chen, M. Teshome, B. Menegaz, H. Javaid, C. Nagi, J. Montalvan, T. Lev, S. Mallya, D. F. Tifrea, R. Edwards, E. Lin, R. Parajuli, S. Hanson, S. Winocour, A. Thompson, B. Lim, D. A. Lawson, K. Kessenbrock, N. Navin, A spatially resolved single-cell genomic atlas of the adult human breast. *Nature* **620**, 181–191 (2023).

24. P. S. Filippou, G. S. Karagiannis, A. Constantinidou, Midkine (MDK) growth factor: A key player in cancer progression and a promising therapeutic target. *Oncogene* **39**, 2040–2054 (2020).

25. S. J. Mentch, M. Mehrmohamadi, L. Huang, X. Liu, D. Gupta, D. Mattocks, P. Gomez Padilla, G. Ables, M. M. Bamman, A. E. Thalacker-Mercer, S. N. Nichenametla, J. W.

Locasale, Histone methylation dynamics and gene regulation occur through the sensing of one-carbon metabolism. *Cell Metab.* **22**, 861–873 (2015).

26. R. Possemato, K. M. Marks, Y. D. Shaul, M. E. Pacold, D. Kim, K. Birsoy, S. Sethumadhavan, H.-K. Woo, H. G. Jang, A. K. Jha, W. W. Chen, F. G. Barrett, N. Stransky, Z.-Y. Tsun, G. S. Cowley, J. Barretina, N. Y. Kalaany, P. P. Hsu, K. Ottina, A. M. Chan, B. Yuan, L. A. Garraway, D. E. Root, M. Mino-Kenudson, E. F. Brachtel, E. M. Driggers, D. M. Sabatini, Functional genomics reveal that the serine synthesis pathway is essential in breast cancer. *Nature* **476**, 346–350 (2011).

27. P. M. Tedeschi, E. K. Markert, M. Gounder, H. Lin, D. Dvorzhinski, S. C. Dolfi, L. L. Y. Chan, J. Qiu, R. S. DiPaola, K. M. Hirshfield, L. G. Boros, J. R. Bertino, Z. N. Oltvai, A. Vazquez, Contribution of serine, folate and glycine metabolism to the ATP, NADPH and purine requirements of cancer cells. *Cell Death Dis.* **4**, –e877 (2013).

28. J. W. Locasale, A. R. Grassian, T. Melman, C. A. Lyssiotis, K. R. Mattaini, A. J. Bass, G. Heffron, C. M. Metallo, T. Muranen, H. Sharfi, A. T. Sasaki, D. Anastasiou, E. Mullarky, N. I. Vokes, M. Sasaki, R. Beroukhim, G. Stephanopoulos, A. H. Ligon, M. Meyerson, A. L. Richardson, L. Chin, G. Wagner, J. M. Asara, J. S. Brugge, L. C. Cantley, M. G. vander Heiden, Phosphoglycerate dehydrogenase diverts glycolytic flux and contributes to oncogenesis. *Nat. Genet.* **43**, 869–874 (2011).

29. D. Samanta, Y. Park, S. A. Andrabi, L. M. Shelton, D. M. Gilkes, G. L. Semenza, PHGDH expression is required for mitochondrial redox homeostasis, breast cancer stem cell maintenance, and lung metastasis. *Cancer Res.* **76**, 4430–4442 (2016).

30. S. Gao, A. Ge, S. Xu, Z. You, S. Ning, Y. Zhao, D. Pang, PSAT1 is regulated by ATF4 and enhances cell proliferation via the GSK3β/β-catenin/cyclin D1 signaling pathway in ER-negative breast cancer. *J. Exp. Clin. Cancer Res.* **36**, 179 (2017).

31. X. Li, D. Gracilla, L. Cai, M. Zhang, X. Yu, X. Chen, J. Zhang, X. Long, H. F. Ding, C. Yan, ATF3 promotes the serine synthesis pathway and tumor growth under dietary serine restriction. *Cell Rep.* **36**, 109706 (2021).

32. S. Tanabe, J. O'Brien, K. E. Tollefsen, Y. Kim, V. Chauhan, C. Yauk, E. Huliganga, R. A. Rudel, J. E. Kay, J. S. Helm, D. Beaton, J. Filipovska, I. Sovadinova, N. Garcia-Reyero, A. Mally, S. S. Poulsen, N. Delrue, E. Fritsche, K. Luettich, C. La Rocca, H. Yepiskoposyan, J. Klose, P. H. Danielsen, M. Esterhuizen, N. R. Jacobsen, U. Vogel, T. W. Gant, I. Choi, R. FitzGerald, Reactive oxygen species in the adverse outcome pathway framework: Toward creation of harmonized consensus key events. *Front. Toxicol.* **4**, 887135 (2022).

33. M. Kusi, M. Zand, L.-L. Lin, M. Chen, A. Lopez, C.-L. Lin, C.-M. Wang, N. D. Lucio, N. B. Kirma, J. Ruan, T. H. M. Huang, K. Mitsuya, 2-Hydroxyglutarate destabilizes chromatin regulatory landscape and lineage fidelity to promote cellular heterogeneity. *Cell Rep.* **38**, 110220 (2022).

34. D. Hanahan, Hallmarks of cancer: New dimensions. *Cancer Discov.* **12**, 31–46 (2022).

35. J. Y. Tsang, K. H. Wong, M. W. Lai, M. D. Lacambra, C. W. Ko, S. K. Chan, C. C. Lam, A. M. Yu, P. H. Tan, G. M. Tse, Nerve growth factor receptor (NGFR): A potential marker for specific molecular subtypes of breast cancer. *J. Clin. Pathol.* **66**, 291–296 (2013).

36. E. Tomellini, Y. Touil, C. Lagadec, S. Julien, P. Ostyn, N. Ziental-Gelus, S. Meignan, J. Lengrand, E. Adriaenssens, R. Polakowska, X. Le Bourhis, Nerve growth factor and proNGF simultaneously promote symmetric self-renewal, quiescence, and epithelial to mesenchymal transition to enlarge the breast cancer stem cell compartment. *Stem Cells* **33**, 342–353 (2015).

37. R. Q. Li, X. H. Zhao, Q. Zhu, T. Liu, H. Hondermarck, R. F. Thorne, X. D. Zhang, J. N. Gao, Exploring neurotransmitters and their receptors for breast cancer prevention and treatment. *Theranostics* **13**, 1109–1129 (2023).

38. P. Jezequel, O. Kerdraon, H. Hondermarck, C. Guerin-Charbonnel, H. Lasla, W. Gouraud, J. L. Canon, A. Gombos, F. Dalenc, S. Delaloge, J. Lemonnier, D. Loussouarn, V. Verriele, M. Campone, Identification of three subtypes of triple-negative breast cancer with potential therapeutic implications. *Breast Cancer Res.* **21**, 65 (2019).

39. Y. Cao, Tumorigenesis as a process of gradual loss of original cell identity and gain of properties of neural precursor/progenitor cells. *Cell Biosci.* **7**, 61 (2017).

40. Y. Cao, Neural is fundamental: Neural stemness as the ground state of cell tumorigenicity and differentiation potential. *Stem Cell Rev. Rep.* **18**, 37–55 (2022).

41. L. Xu, M. Zhang, L. Shi, X. Yang, L. Chen, N. Cao, A. Lei, Y. Cao, Neural stemness contributes to cell tumorigenicity. *Cell Biosci.* **11**, 21 (2021).

42. D. Hanahan, M. Monje, Cancer hallmarks intersect with neuroscience in the tumor microenvironment. *Cancer Cell* **41**, 573–580 (2023).

43. W. Wang, L. Li, N. Chen, C. Niu, Z. Li, J. Hu, J. Cui, Nerves in the tumor microenvironment: Origin and effects. *Front. Cell Dev. Biol.* **8**, 601738 (2020).

44. M. J. Mahendralingam, H. Kim, C. W. McCloskey, K. Aliar, A. E. Casey, P. Tharmapalan, D. Pellacani, V. Ignatchenko, M. Garcia-Valero, L. Palomero, A. Sinha, J. Cruickshank, R. Shetty, R. N. Vellanki, M. Koritzinsky, V. Stambolic, M. Alam, A. D. Schimmer, H. K. Berman, C. J. Eaves, M. A. Pujana, T. Kislinger, R. Khokha, Mammary epithelial cells have lineage-rooted metabolic identities. *Nat. Metab.* **3**, 665–681 (2021).

45. N. Kannan, L. V. Nguyen, M. Makarem, Y. Dong, K. Shih, P. Eirew, A. Raouf, J. T. Emerman, C. J. Eaves, Glutathione-dependent and -independent oxidative stress-control mechanisms distinguish normal human mammary epithelial cell subsets. *Proc. Natl. Acad. Sci. U.S.A.* **111**, 7789–7794 (2014).

46. W. Guo, J. K. Choi, J. L. Kirkland, B. E. Corkey, J. A. Hamilton, Esterification of free fatty acids in adipocytes: A comparison between octanoate and oleate. *Biochem. J.* **349**, 463–471 (2000).

47. J. B. Lockridge, M. L. Sailors, D. J. Durgan, O. Egbejimi, W. J. Jeong, M. S. Bray, W. C. Stanley, M. E. Young, Bioinformatic profiling of the transcriptional response of adult rat cardiomyocytes to distinct fatty acids. *J. Lipid Res.* **49**, 1395–1408 (2008).

48. S. Balaban, R. F. Shearer, L. S. Lee, M. van Geldermalsen, M. Schreuder, H. C. Shtein, R. Cairns, K. C. Thomas, D. J. Fazakerley, T. Grewal, J. Holst, D. N. Saunders, A. J. Hoy,

Adipocyte lipolysis links obesity to breast cancer growth: Adipocyte-derived fatty acids drive breast cancer cell proliferation and migration. *Cancer Metab.* **5**, 1 (2017).

49. Y. Liang, H. Han, L. Liu, Y. Duan, X. Yang, C. Ma, Y. Zhu, J. Han, X. Li, Y. Chen, CD36 plays a critical role in proliferation, migration and tamoxifen-inhibited growth of ER-positive breast cancer cells. *Oncogenesis* **7**, 98 (2018).

50. J. Gyamfi, J. H. Yeo, D. Kwon, B. S. Min, Y. J. Cha, J. S. Koo, J. Jeong, J. Lee, J. Choi, Interaction between CD36 and FABP4 modulates adipocyte-induced fatty acid import and metabolism in breast cancer. *NPJ Breast Cancer* **7**, 129 (2021).

51. M. Freed, P. Storey, A. A. Lewin, J. Babb, M. Moccaldi, L. Moy, S. G. Kim, Evaluation of breast lipid composition in patients with benign tissue and cancer by using multiple gradient-echo MR imaging. *Radiology* **281**, 43–53 (2016).

52. G. Mamalakis, C. Hatzis, E. de Bree, E. Sanidas, D. D. Tsiftsis, J. Askoxylakis, M. Daskalakis, G. Tsibinos, A. Kafatos, Adipose tissue fatty acids in breast cancer patients versus healthy control women from Crete. *Ann. Nutr. Metab.* **54**, 275–282 (2009).

53. Y. Zhu, M. D. Aupperlee, Y. Zhao, Y. S. Tan, E. L. Kirk, X. Sun, M. A. Troester, R. C. Schwartz, S. Z. Haslam, Pubertal and adult windows of susceptibility to a high animal fat diet in Trp53-null mammary tumorigenesis. *Oncotarget* **7**, 83409–83423 (2016).

54. E. Mullarky, N. C. Lucki, R. Beheshti Zavareh, J. L. Anglin, A. P. Gomes, B. N. Nicolay, J. C. Wong, S. Christen, H. Takahashi, P. K. Singh, J. Blenis, J. D. Warren, S. M. Fendt, J. M. Asara, G. M. DeNicola, C. A. Lyssiotis, L. L. Lairson, L. C. Cantley, Identification of a small molecule inhibitor of 3-phosphoglycerate dehydrogenase to target serine biosynthesis in cancers. *Proc. Natl. Acad. Sci. U.S.A.* **113**, 1778–1783 (2016).

55. R. J. Blaschke, A. R. Howlett, P. Y. Desprez, O. W. Petersen, M. J. Bissell, Cell differentiation by extracellular matrix components. *Methods Enzymol.* **245**, 535–556 (1994).

56. M. Yuan, D. M. Kremer, H. Huang, S. B. Breitkopf, I. Ben-Sahra, B. D. Manning, C. A. Lyssiotis, J. M. Asara, Ex vivo and in vivo stable isotope labelling of central carbon

metabolism and related pathways with analysis by LC–MS/MS. *Nat. Protoc.* **14**, 313–330 (2019).

57. D. Kraft, A software package for sequential quadratic programming. *Forschungsbericht-Deutsche Forschungs- und Versuchsanstalt fur Luft- und Raumfahrt ["Research report-German Aerospace Research and Testing Institute"]* (1988).

58. W. M. Oldham, J. Loscalzo, Quantification of 2-hydroxyglutarate enantiomers by liquid chromatography-mass spectrometry. *Bio Protoc.* **6**, e1908 (2016).

59. B. M. Gyori, G. Venkatachalam, P. S. Thiagarajan, D. Hsu, M.-V. Clement, OpenComet: An automated tool for comet assay image analysis. *Redox Biol.* **2**, 457–465 (2014).

60. K. J. Livak, T. D. Schmittgen, Analysis of relative gene expression data using real-time quantitative PCR and the $2^{-\Delta\Delta C}{}_{T}$ method. *Methods* **25**, 402–408 (2001).

61. T. Stuart, A. Butler, P. Hoffman, C. Hafemeister, E. Papalexi, W. M. Mauck, Y. Hao, M. Stoeckius, P. Smibert, R. Satija, Comprehensive integration of single-cell data. *Cell* **177**, 1888–1902.e21 (2019).

62. C. S. McGinnis, D. M. Patterson, J. Winkler, D. N. Conrad, M. Y. Hein, V. Srivastava, J. L. Hu, L. M. Murrow, J. S. Weissman, Z. Werb, E. D. Chow, Z. J. Gartner, MULTI-seq: Sample multiplexing for single-cell RNA sequencing using lipid-tagged indices. *Nat. Methods* **16**, 619–626 (2019).

63. I. Korsunsky, N. Millard, J. Fan, K. Slowikowski, F. Zhang, K. Wei, Y. Baglaenko, M. Brenner, P. R. Loh, S. Raychaudhuri, Fast, sensitive and accurate integration of single-cell data with Harmony. *Nat. Methods* **16**, 1289–1296 (2019).

64. D. Aran, A. P. Looney, L. Liu, E. Wu, V. Fong, A. Hsu, S. Chak, R. P. Naikawadi, P. J. Wolters, A. R. Abate, A. J. Butte, M. Bhattacharya, Reference-based analysis of lung single-cell sequencing reveals a transitional profibrotic macrophage. *Nat. Immunol.* **20**, 163–172 (2019).

65. A. D. Reed, S. Pensa, A. Steif, J. Stenning, D. J. Kunz, L. J. Porter, K. Hua, P. He, A.-J. Twigger, A. J. Q. Siu, K. Kania, R. Barrow-McGee, I. Goulding, J. J. Gomm, V. Speirs, J. L.

Jones, J. C. Marioni, W. T. Khaled, A single-cell atlas enables mapping of homeostatic cellular shifts in the adult human breast. *Nat. Genet.* **56**, 652–662 (2024).

66. J. A. Bibby, D. Agarwal, T. Freiwald, N. Kunz, N. S. Merle, E. E. West, P. Singh, A. Larochelle, F. Chinian, S. Mukherjee, B. Afzali, C. Kemper, N. R. Zhang, Systematic single-cell pathway analysis to characterize early T cell activation. *Cell Rep.* **41**, 111697 (2022).

67. S. Jin, C. F. Guerrero-Juarez, L. Zhang, I. Chang, R. Ramos, C.-H. Kuan, P. Myung, M. V. Plikus, Q. Nie, Inference and analysis of cell-cell communication using CellChat. *Nat. Commun.* **12**, 1088 (2021).

68. A. Wagner, C. Wang, J. Fessler, D. DeTomaso, J. Avila-Pacheco, J. Kaminski,S. Zaghouani, E. Christian, P. Thakore, B. Schellhaass, E. Akama-Garren, K. Pierce,V. Singh, N. Ron-Harel, V. P. Douglas, L. Bod, A. Schnell, D. Puleston, R. A. Sobel,M. Haigis, E. L. Pearce, M. Soleimani, C. Clish, A. Regev, V. K. Kuchroo, N. Yosef, Metabolic modeling of single Th17 cells reveals regulators of autoimmunity. *Cell* **184**, 4168–4185.e21 (2021).

69. W. Zhou, T. J. Triche Jr., P. W. Laird, H. Shen, SeSAMe: Reducing artifactual detection of DNA methylation by Infinium BeadChips in genomic deletions. *Nucleic Acids Res.* **46**, e123 (2018).
